# Visualizing dynamics of membrane rafts on live cells

**DOI:** 10.1126/sciadv.adv7001

**Published:** 2025-11-28

**Authors:** Hsiang-Ling Chuang, Yu-Chen Fa, Kum-Yi Cheng, Er-Chien Horng, Yi-Te Chou, Richard P. Cheng, Li-Chen Wu, Ja-an Annie Ho, Chun-hsien Chen

**Affiliations:** ^1^ Department of Chemistry, National Taiwan University, Taipei, Taiwan.; ^2^ Center for Emerging Material and Advanced Devices, National Taiwan University, Taipei, Taiwan.; ^3^ Department of Biochemical Science and Technology, National Taiwan University, Taipei, Taiwan.; ^4^ Department of Applied Chemistry, National Chi Nan University, Puli, Nantou, Taiwan.; ^5^ Center for Biotechnology, National Taiwan University, Taipei, Taiwan.

## Abstract

Membrane rafts are cellular portals to external stimuli that trigger signaling cascades for sophisticated yet remarkable biochemical activities. Visualization of the topographic evolution of membrane rafts remains unreported on live cells due to the nanosized and dynamic nature. Here, an imaging strategy involving atomic force microscopy and Hadamard product is developed to unveil membrane-raft features. Michigan Cancer Foundation-7 (MCF-7) cells were subjected to fibrinogen or manganese(II) (Mn^2+^)/resveratrol, both of which are ligands of integrin α_V_β_3_ embedded within membrane rafts; the former promotes metastasis, and the latter enables apoptosis. MCF-7 cellular membranes responded to the two stimulants markedly different. The size, height, spatiotemporal trajectory, and persistent time of ligand-activated nanodomains are revealed. This approach opens up a visualized platform toward the understanding of activation-associated signaling cascades.

## Teaser

The size, height, trajectory, and duration of activated integrins—key portals in cell signaling—are visualized on live cells.

## INTRODUCTION

The cell membrane gates extracellular stimuli, which drive membrane protein trafficking (*1*, *2*) and trigger signaling cascades (*3*, *4*), leading to subsequent cellular processes (*5*). The membrane raft hypothesis by Simons and Ikonen (*6*, *7*) postulates the involvement of heterogeneous self-organized nano-sized membrane domains containing specific lipid-protein and protein-protein interactions in such gating (*8*, *9*). Accordingly, understanding the nanoscale dynamics of membrane rafts is one of the first steps to delineate the corresponding behavioral patterns and to comprehend fundamental cellular mechanisms. The membrane raft nanodomains are abundant in cholesterol and saturated lipids (such as sphingolipids), ceramide (*10*, *11*), and proteins, exhibiting a liquid crystalline bilayer [termed liquid-ordered phases (L_o_)], whereas the surroundings contain less cholesterol to form liquid-disordered phases (L_d_) (*7*). Many cellular mechanisms implicated in the raft hypothesis would be unveiled by direct observation of the lateral heterogeneity with spatiotemporal information. Membrane rafts are widely accepted to be 10 to 200 nm (*12*) based on extensive studies on reconstituted model membranes extracted from cells (*13*, *14*). Conventional optical microscopic approaches to visualize the membrane dynamics remain a great challenge because the required resolution is smaller than the Abbe diffraction limit of ~200 nm. Because of the lack of topographic images demonstrating distinct nanoscopic features portrayed in the raft hypothesis, the very existence of rafts has been under active debate (*15*). Efforts to resolve this controversy have involved emerging spectroscopies or microscopies and advances in spatiotemporal resolution (*16*). For example, the use of extrinsic fluorescent molecules casts doubts on relevant measurements due to unexpected interactions of these molecules with the raft components. To this end, recent super-resolution fluorescence microscopic (SRFM) studies on live cells have enabled the direct observation of membrane rafts using two types of dyes independently. One type is associated with the localization of cholesterol and reveals the membrane heterogeneity of the nanodomains < 40 nm (*17*). Contreras and co-workers (*17*) developed a sterol-derivatized fluorescently labeled probe with a spacer between the sterol moiety and the dye to preserve membrane integrity. Kemmoku *et al.* (*18*) visualized heterogeneously distributed cholesterol-rich domains by iD4H, a cholesterol sensor produced from *Clostridium perfringens* theta-toxin (*19*). The other type is sensitive to the polarity of local environments, such as Laurdan (*17*) and di-4-ANEPPDHQ (*18*). Their emission wavelengths depend on the membrane lateral packing, validating no SRFM probe-induced interchange between L_o_ and L_d_ phases. Alternatively, deuterated lipids behave indistinguishably from the pristine ones, yet enable in vivo small-angle neutron scattering (SANS) measurements. Nickels *et al.* (*20*) studied a Gram-positive bacterium *Bacillus subtilis* by SANS and determined a domain size of <40 nm for membrane rafts, providing one of the first glimpses for the membrane raft size without any potential perturbation by fluorescent molecules. Currently, no single technique can escape its inherit methodological weakness, but the strengths of each approach can provide important aspects and collectively advance our understanding of membrane rafts (*16*, *21*). Nonetheless, it is still highly desirable to develop imaging tools that require minimal experimental preparation and confer self-explanatory images with the morphological dynamics of membrane rafts on live cells. Currently, membrane raft height has only been estimated in cryo–electron microscopy (cryo-EM) experiments (*22*, *23*), but membrane raft persistent time (i.e., lifetime) has yet to be estimated in any capacity based on experimental observations.

**Fig. 1.**
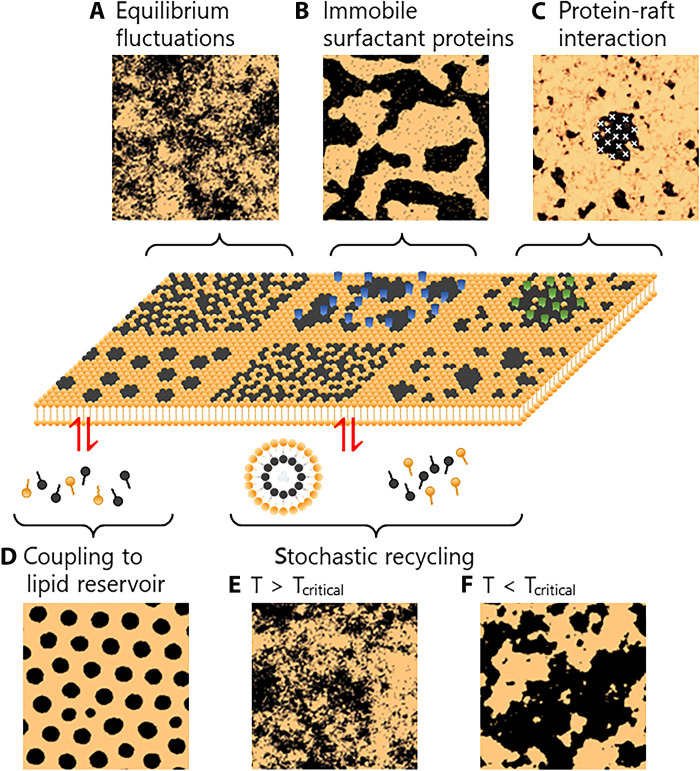
Illustration of membrane rafts organized by theoretical mechanisms. The snapshots were calculated by concisely denotative physical models of (**A**) transient compositions due to thermal fluctuations around the critical point (T = T_critical_) (*26*, *63*), (**B**) pinning by immobile membrane proteins (*26*), (**C**) strongly clustered proteins (*27*), (**D**) coupling of the membrane with cytoplasmic compositions (*26*, *64*), stochastic recycling of lipids at (**E**) miscible (T > T_critical_) (*26*), and (**F**) immiscible (T < T_critical_) (*26*, *27*) compositions. The membrane rafts (i.e., the L_o_ phase) are colored in black. The top row represents thermal equilibrium processes in which the thermodynamic forces dictated the formation and structures of membrane rafts (*28*). The snapshots in the bottom row were calculated by nonequilibrium cellular processes involving interactions of the membrane with the interior of the cell (*26*). For details, please see a review article by Fan *et al.* (*26*) from which Fig. 1 is adapted and modified.

Theoretical modeling can be viewed as microscopy in silico (*24*, *25*), enabling the visualization of plasma membranes with unparalleled spatiotemporal details compared to the finest available experimental techniques. Modeling is effective in characterizing raft size, lateral organization, and spatial distribution to explain raft related processes. For example, Fig. 1 displays simulation results summarized and illustrated by Fan *et al.* (*26*–*28*). Denoted above the snapshot panels are the physical models. These models were mainly based on a nonlinear diffusion equation with stochastic Gaussian noise for the lipids within the exoplasmic leaflet (*28*). Panel A was calculated for membranes under thermodynamic equilibrium near the miscibility critical point (T_critical_), in which lateral heterogeneity was driven by thermal noise. Postulations for panels B and C were the presence of immobile proteins and attractive interactions between rafts, respectively. Panels D to F were calculated in which membrane components recycled to the cytoplasm. The distribution patterns were somewhat discernible, providing a rough blueprint for the different raft formation mechanisms. On the basis of these patterns, experimental topographic images may be used to determine the operating mechanism.

Atomic force microscopy (AFM) can be used to obtain topographic information about cell membranes, enabling direct imaging under physiological environments (*29*–*32*) with nanometer-level spatial resolution. Typical AFM sample preparation is relatively simple without the need of fixing, staining, or labeling. However, the lack of chemical specificity makes the assignment and interpretation of the morphological details very challenging. Given the presence of thousands of lipid types and proteins in the cell membrane, the images exhibit overwhelming amounts of uncharacterizable and indistinguishable components. Hence, the heavily loaded information is unexplainable by analyzing the morphology alone. To reduce the complexity of membrane rafts, AFM imaging has been carried out on reconstituted cell membranes (*33*) or artificial model membranes (*14*, *33*), composed of cholesterol and limited types of lipids, such as 1,2-dipalmitoyl-*sn*-glycero-3-phosphocholine, dioleoylphosphatidylcholine, and 1-palmitoyl-2-oleoyl-*sn*-glycero-3-phosphochline. These protocols allow unequivocal interpretation of the AFM images but at the loss of membrane proteins and in the absence of cellular bioactivities. Here, we present an AFM study of membrane rafts on live cells. AFM can simultaneously probe spatial information about the topography and mechanical stiffness of the cell surface (*29*, *30*, *34*). To better comprehend the images, the higher and stiffer characteristics of membrane rafts are accentuated by the operation of the Hadamard product (vide infra), which suppresses nonmembrane raft features (e.g., higher/softer and lower/stiffer). To remedy the chemical specificity shortcoming of AFM, we administered stimulants such as fibrinogen (*35*) and Mn^2+^/resveratrol (*36*, *37*) to live Michigan Cancer Foundation-7 (MCF-7) cells to bind and activate integrins, transmembrane cell surface receptors that transduce biochemical and mechanical signals between the extracellular matrix and cytoskeleton (*38*). Fibrinogen binds to activated and unactivated integrins (α_V_β_3_ or α_5_β_1_) (*38*–*45*), whereas resveratrol binds to Mn^2+^-activated integrin α_V_β_3_, which most likely resides within the membrane raft (*46*–*48*). Hence, the downstream signaling pathways should be very different (*36*–*38*). Accordingly, the corresponding changes in size and height in the AFM images may be attributed to integrin-containing membrane raft dynamics. The AFM images resemble some of the patterns presented in Fig. 1, suggesting a strong correlation of the stimulant-triggered mechanisms to the simulation-based models.

## RESULTS

### Identification of membrane rafts on cell surface by AFM

**Fig. 2.**
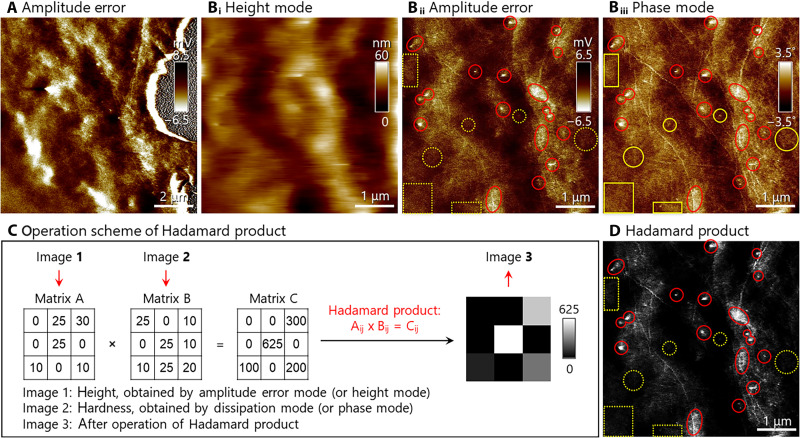
Raw images of a live MCF-7 cell and the operation of Hadamard product to manifest features higher and stiffer than their surroundings. (**A**) Image at the cell edge. The gray semicircle at the top right was the petri dish substrate. (**B**_**i**_ to **B**_**iii**_) Synchronically acquired FastScan AFM images at the same location by (B_i_) height mode, (B_ii_) amplitude error, and (B_iii_) phase mode. (**C**) Operation scheme of Hadamard product. (**D**) Image after the operation of Hadamard product for raw data of (B_ii_) and (B_iii_). Red circles exemplify high and stiff features, while the yellow ones are either high but soft or stiff but low. Conditions: solution: phosphate-buffered saline (PBS) (10 mM phosphate, pH 7.4); scan rate: 51 s per frame; and drive frequency: 110 to 130 kHz. Image size: (A) 10 μm by 10 μm and [(B) and (D)] 5 μm by 5 μm.

Live MCF-7 cells were interrogated by FastScan AFM, which enabled the tracking of membrane raft motions with a temporal resolution of ~30 s per frame, about six- to 20-fold faster than that of typical AFM imaging with comparable quality. Limited by the specifications of our instrument, the largest imaging area was 36 μm by 36 μm, and hence, a complete MCF-7 cell could not fit into one single image. Other AFM microscopes can typically scan over a square of 90 μm by 90 μm, available for viewing whole cells (e.g., fig. S1) but cannot perform fast scan imaging. AFM imaging modes pertinent to this study are succinctly described in the Supplementary Materials. In fig. S2, the cytoskeleton went from upper left to lower right and was dynamically drifting and restructuring. For Fig. 2B (_i_ to _iii_), multiple modes were operated simultaneously over the same cellular location, which was zoomed in for Fig. 2A. Both height mode (Fig. 2B_i_) and amplitude error (Fig. 2B_ii_) yielded topographic information. Amplitude error (in millivolts) showed higher spatial resolution, although the imaging mechanism granted height mode (in nanometers) straightforward interpretation of the surface morphology. The phase angles of phase mode images (Fig. 2B_iii_) indicated different degrees of surface hardness. In principle, the advanced or lagged degrees in phase cannot be specifically assigned to a hard or soft surface. Nonetheless, the shape of the cytoskeletons could be readily recognized, and the response in phase would be the benchmark for a hard surface. In the case of Fig. 2B_iii_, the cytoskeleton appeared phase advanced, and thus features with advanced and lagged phases were considered relatively hard and soft, respectively. We deposit in the Supplementary Materials a set of images (fig. S1) acquired by PeakForce QNM equipped with a 90-μm scanner. The results provided unambiguous quantitative information of surface hardness, although the image quality and scan rate were insufficient to track the dynamics on the cellular membrane.

Sterol- and sphingolipid-enriched domains (*12*) are higher and stiffer than the surrounding membrane. Accordingly, features that appeared higher in Fig. 2B_ii_ and stiffer in Fig. 2B_iii_ were considered to include membrane rafts, although contributions from other nanodomains are not excluded. Membrane rafts are elevated features compared to the surrounding membrane (*49*). Furthermore, membrane rafts correspond to L_o_ domains (*5*, *50*), which are stiffer compared to surrounding L_d_ domains (*51*). Therefore, to pinpoint membrane rafts, the Hadamard product images were generated to accentuate only regions that are simultaneously higher and stiffer. The red circles mark examples that are both higher and stiffer (i.e., membrane rafts). The yellow circles mark examples where the features are higher but softer or stiffer but not elevated from their surroundings (Fig. 2D and fig. S1E). Although cytoskeletons are also higher and stiffer, their elongated shapes are discernible from rafts (fig. S2). The Hadamard image obtained by combining the amplitude error (height) and phase mode (hardness) enabled facile identification of features resembling membrane rafts. This assignment was further supported by the response toward administered fibrinogen and Mn^2+^/resveratrol (vide infra).

### Response of membrane rafts to fibrinogen and Mn^2+^/resveratrol

**Fig. 3.**
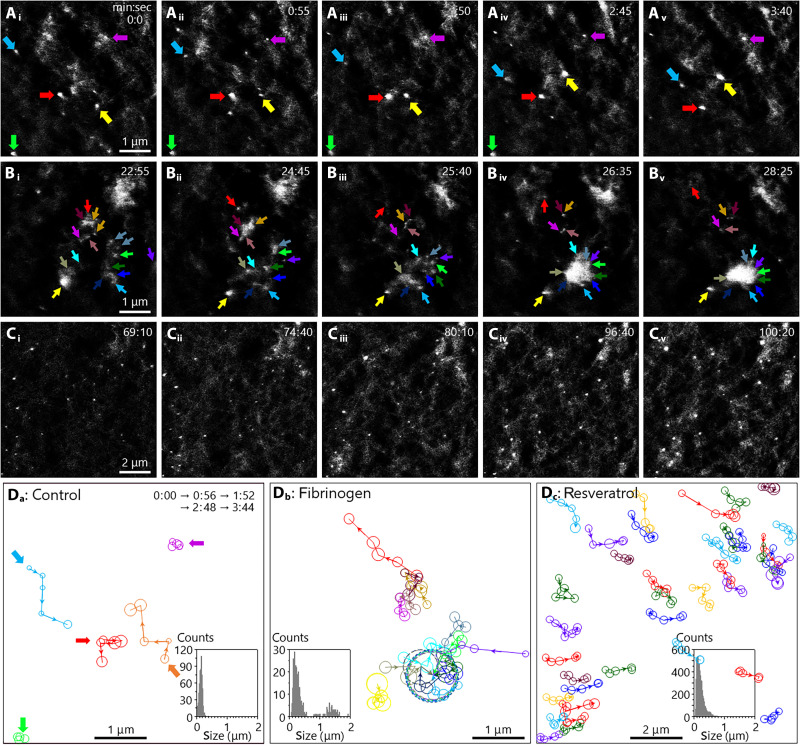
Dynamics of membrane rafts on live MCF-7 cells. Experiments were carried out in the environments of (**A**) blank phosphate buffer and additional treatment with (**B**) fibrinogen and (**C**) Mn^2+^/resveratrol. (**D**) Trajectories of membrane rafts. In (D_a_) to (D_c_), the circle diameters and arrows correspond to the sizes and moving directions of the rafts, respectively. The histograms and table S1 summarize the size distribution. In (D_b_) the 1-μm circles (gray) indicate the fused aggregate. Solution: (A) PBS (10 mM phosphate, pH 7.4), (B) PBS with 70 μM fibrinogen, and (C) PBS with 50 μM Mn^2+^ and 10 μM resveratrol. Scan rate: 28 s per frame. Image size: [(A) and (B)] 5 μm by 5 μm and (C) 10 μm by 10 μm. Other imaging conditions were the same as those of Fig. 2.

**Fig. 4.**
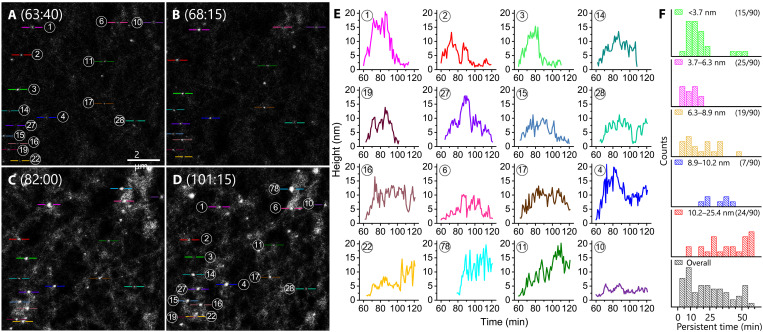
Progression of topographic heights of resveratrol-treated membrane rafts. (**A** to **D**) Images were obtained from the same experiment as those in Fig. 3C. Data collection started immediately after the resveratrol solution was added into the AFM liquid cell. The images were taken after 60 min because of the ~1 hour required to activate integrins α_V_β_3_ by Mn^2+^ (fig. S8). (**E**) Examples of the height evolution for the protrusions. The height information was measured from the tapping mode deflection images. Some features did not appear at the beginning (e.g., 27 and 78) or in the end (e.g., 1, 3, 14, and 19) of the observation, and their data were plotted accordingly. Movie S5 presents the evolution in height for four features. (**F**) Histograms of height-dependent persistent time, which is defined as the duration during which the height remains above 3.7 nm (for detail, see fig. S7). Solution: PBS with 50 μM Mn^2+^ and 10 μM resveratrol. Scan rate: 28 s per frame. Image size: 10 μm by 10 μm. Other imaging conditions were the same as those of Fig. 2B.

The trajectory and height evolution of membrane rafts were monitored in the absence (Fig. 3A, movie S2, and fig. S3) and presence of stimulants (Figs. 3, B and C, and 4; movies S3 and S4; and figs. S4 and S5). In the absence of stimulus, the nominal diameter and height of membrane rafts were about 150 and 1.9 nm (fig. S6), respectively. This size is consistent with the current estimation of 10 to 200 nm described in the introductory section for membrane rafts (*52*). Several membrane rafts in Fig. 3 (A_i_ to A_v_) were monitored (Fig. 3D_a_), showing a variety of motions. Within 4 min, the rafts indicated by blue and yellow arrows traveled further than 1 μm in opposite directions. The two rafts indicated by purple and green arrows appeared stationary.

The cells were imaged upon administering fibrinogen; images representing 23rd to 29th min after administering are presented in Fig. 3B. The ~0.8-μm–sized feature (in the top right corner of Fig. 3B_i_) fluctuated in size and eventually dissipated. Small spots indicated by arrows (in the bottom right part of Fig. 3B_i_) conglomerated and turned into ~1 μm in size and ~45 nm in height entity. The conglomerated features such as the ~0.8-μm and ~1-μm ones suggested that individual rafts (10 to 200 nm) in Fig. 3B_iii_ could not be resolved in the features (~0.8 and ~1 μm) of Fig. 3B_iv_. This phenomenon was observed in 18 of 22 cells (fig. S11). The ligand-activated changes were absent in roughly 20% of the cells, most likely due to the lack of receptors in the section chosen to be monitored.

The cells were imaged upon exposure to resveratrol in the presence of Mn^2+^ (Fig. 3C and fig. S12). Images were acquired after 1 hour to ensure activation of α_V_β_3_ by Mn^2+^ (please see confocal images in figs. S8 and S13, which includes representative images of 27 cells in total). Moreover, confocal microscope characterization (fig. S10) demonstrates that α_V_β_3_ localizes to C-Laurdan–defined liquid-ordered domains (membrane rafts). The protrusions increased in number and grew slightly larger and higher (Fig. 4). In the top right and bottom left corners of the images, there were elevated terraces higher than the background membrane. In the terraces, individual protrusions were distinctly delineated, remaining discrete rather than merging, unlike the conglomerated features observed upon exposure to fibrinogen (e.g., Fig. 3, B_iv_ and B_v_). To include the features in the top right and bottom left corners in one frame, the image size was twice of those in Fig. 3 (A and B).

The height of the integrin membrane rafts upon Mn^2+^ activation in the presence of resveratrol was monitored (Fig. 4 and movie S5). In the absence of either Mn^2+^ activation or resveratrol, the rafts were ~1.9 nm higher than the background membrane (fig. S6), consistent with theoretical modeling (*53*) and AFM studies on reassembled rafts that were isolated and purified from cell membranes (*33*). After the introduction of both Mn^2+^ and resveratrol, the protruded features were tracked. In a representative experiment (Fig. 4), about 28% of the protrusions did not respond, and the duration for their presence lasted 10 to 20 min (i.e., the heights returned to the background level; fig. S7). Another ~31% of the protrusions showed heights of more than 10 nm (Fig. 4E), consistent with a fully extended α_V_β_3_ integrin based on studies by simulations (*54*, *55*), biomembrane force probe (*56*), and cryo-EM (*23*). The rest ~41% was activated, but the maximal height was not higher than 10 nm. For the membrane rafts with heights corresponding to fully extended integrins, about 65% of the stimulated events completed in the 90th to 120th min, and about 35% continued after the 120th min (Fig. 4E). The persistent time (defined on page 3 of Supplementary Text and fig. S7A) broadly spread from 10 to 60 min (Fig. 4F), providing the information about the lifetime of a resveratrol-bound, Mn^2+^ activated integrin.

## DISCUSSION

### Biochemical implications

An important prospect of visualizing stimulant-triggered membrane dynamics is to guide, with the support of theoretical modeling, the exploration of downstream signaling pathways beneath the outer exoplasmic leaflet. The AFM images of MCF-7 cells upon administering fibrinogen and Mn^2+^/resveratrol (Fig. 3, B and C) resembled the simulated patterns in Fig. 1 (C and D), respectively, suggesting mechanisms associated with attractive protein-raft interactions (*28*) for the former and membrane components recycling to the cytoplasm (*28*) for the latter. In these cases, the pure physical models appeared to be able to correspond to specific biochemical processes. The unbound bent integrin β_3_ chain reorients and stretches upon binding of the β_3_ cytoplasmic tail to talin and kindlin (*57*). The subsequent binding to fibrinogen converts the integrin to a fully activated extended open conformation. Alternatively, ligands (e.g., fibrinogen) in the extracellular matrix can activate unbound bent integrins directly without the assistance of talin binding (*58*). The fibrinogen-bound activated integrins can then recruit signaling regulators, such as vinculin, which reinforces the β subunit–talin–actomyosin interaction and facilitates integrin clustering (*59*), equivalent to the attractive protein-raft interactions in Fig. 1C.

The AFM images of MCF-7 cells in the presence of Mn^2+^/resveratrol bear resemblance to Fig. 1D. The corresponding physical model suggests recycling of small molecules between the cell membrane and cytosol. The added Mn^2+^/resveratrol interacts with the exposed β_3_ domain (*36*), which activates the integrin and downstream molecules including Rac–guanosine triphosphatase (*60*). This recruits phospholipase C from the cytosol to cell membrane (*61*) and elicits the breakdown of phosphatidylinositol 4,5-bisphosphate (PIP_2_) to inositol triphosphate (IP_3_) and diacylglycerol. The IP_3_ is released to the cytosol in exchange with phosphatidylinositol, which is synthesized in the endoplasmic reticulum and then delivered to the cell membrane to reform PIP_2_ (*62*). In either the fibrinogen or the Mn^2+^/resveratrol case, we cannot completely rule out the possibility that the activated integrins may be bound to the actin cytoskeleton.

In summary, this study manifests the strengths of AFM in the synchronous acquisition of topographic and hardness information to generate the corresponding Hadamard image. These Hadamard images enable the facile identification of membrane rafts, providing temporal information about location, movement, size, and height. Specifically, this study provides an estimation for the persistent time of an activated integrin. Equipped with these Hadamard images with the distribution and outlines of membrane rafts, it is therefore feasible to examine the computational snapshots and identify the interrelated physical models to guide subsequent studies on downstream signal transduction pathways.

## MATERIALS AND METHODS

### Chemicals and solutions

A phosphate-buffered saline (PBS) solution containing 1.0 mM Hepes (C_8_H_18_N_2_O_4_S, Sigma-Aldrich) and 10% (v/v) fetal bovine serum (FBS; Gibco) was used for the AFM imaging of live MCF-7 cells in control experiments without stimulants. For experiments involving the activated integrins, the solutions of the stimulants were prepared in PBS. Specifically, the solutions were 0.14 mM fibrinogen (Sigma-Aldrich) or 20 μM resveratrol (≥ 99%; high-performance liquid chromatography grade, Sigma-Aldrich) in PBS composed of 1.0 mM Hepes and 10% FBS. The resveratrol solution also contained 100 μM MnSO_4_ (BioReagent grade, Sigma-Aldrich). For the control experiments, the AFM liquid cell (a custom-made petri dish; Alpha Plus Scientific Corp., Taoyuan City, Taiwan) contained cultured MCF-7 cells and 5.0 ml of PBS. For the subsequent stimulation, another 5 ml of the stimulant solution was added to make a 10-ml solution of 70 μM fibrinogen or 10 μM resveratrol with 50 μM MnSO_4_.

### Cell culture

The MCF-7 human breast cancer cell line (HTB-22TM, American Type Culture Collection) was purchased from the Bioresource Collection and Research Center (Hsinchu, Taiwan). Cells were cultured in Dulbecco’s modified Eagle’s medium (12800017, Gibco, Waltham, MA, USA) supplemented with 10% FBS, 1% penicillin-streptomycin (SV30010, HyClone, Logan, UT, USA), and 10 mM Hepes. The culture was maintained in a humidified incubation system with 5% CO_2_ at 37°C. The growth medium was refreshed every 48 hours, and cells were trypsinized using 0.1% trypsin (SH30042.01, HyClone, Logan, UT, USA) and subcultured upon reaching ~90% confluence. For AFM imaging, 8 × 10^4^ MCF-7 cells per well were seeded and incubated in a custom-made petri dish in a 5% CO_2_ incubator at 37°C for 48 hours before studying.

### AFM imaging on live MCF-7 cells

AFM imaging was performed using a Dimension AFM equipped with FastScan and Icon scanners (Bruker). To preserve the cell viability during AFM observation, the imaging stage was hosted in a vibration-isolation chamber in which the PBS solution was maintained at 37°C (Model 335 temperature controller, Lakeshore). The suitable spring constants of AFM tips for imaging cell membranes by tapping mode (FastScan scanner) and PeakForce QNM Mode (Icon scanner) were found to be 0.25 N/m (FastScan-D, Bruker) and 0.6 N/m (HHQ:NSC36, MikroMasch), respectively. Typical imaging parameters for tapping mode were amplitude setpoints of 60 to 120 mV and scan rates of 10.0 to 18.6 Hz, corresponding to 51.2 to 27.5 s per image frame. For PeakForce QNM Mode, the parameters for amplitude, setpoint force, and scan rates were 100 to 200 nm, 0.10 nN, and 0.4 to 0.6 Hz, respectively.

## Supplementary Materials

### The PDF file includes:

Supplementary Text Figs. S1 to S14 Data S1 Legends for movies S1 to S5 Tables S1 and S2 References

## References

[1] B. Diaz-Rohrer, K. R. Levental, I. Levental, Rafting through traffic: Membrane domains in cellular logistics. Biochim. Biophys. Acta Biomembr. 1838, 3003–3013 (2014). 10.1016/j.bbamem.2014.07.029 25130318

[2] B. B. Diaz-Rohrer, K. R. Levental, K. Simons, I. Levental, Membrane raft association is a determinant of plasma membrane localization. Proc. Natl. Acad. Sci. U.S.A. 111, 8500–8505 (2014). 24912166 10.1073/pnas.1404582111 PMC4060687

[3] K. Simons, D. Toomre, Lipid rafts and signal transduction. Nat. Rev. Mol. Cell Biol. 1, 31–39 (2000). 11413487 10.1038/35036052

[4] A. Kusumi, T. K. Fujiwara, N. Morone, K. J. Yoshida, R. Chadda, M. Xie, R. S. Kasai, K. G. N. Suzuki, Membrane mechanisms for signal transduction: The coupling of the meso-scale raft domains to membrane-skeleton-induced compartments and dynamic protein complexes. Semin. Cell Dev. Biol. 23, 126–144 (2012). 22309841 10.1016/j.semcdb.2012.01.018

[5] E. Sezgin, I. Levental, S. Mayor, C. Eggeling, The mystery of membrane organization: Composition, regulation and roles of lipid rafts. Nat. Rev. Mol. Cell Biol. 18, 361–374 (2017). 28356571 10.1038/nrm.2017.16 PMC5500228

[6] K. Simons, E. Ikonen, Functional rafts in cell membranes. Nature 387, 569–572 (1997). 9177342 10.1038/42408

[7] S. L. Veatch, N. Rogers, A. Decker, S. A. Shelby, The plasma membrane as an adaptable fluid mosaic. Biochim. Biophys. Acta Biomembr. 1865, 184114 (2023). 36581017 10.1016/j.bbamem.2022.184114 PMC9922517

[8] D. Lingwood, K. Simons, Lipid rafts as a membrane-organizing principle. Science 327, 46–50 (2010). 20044567 10.1126/science.1174621

[9] T. Harayama, H. Riezman, Understanding the diversity of membrane lipid composition. Nat. Rev. Mol. Cell Biol. 19, 281–296 (2018). 29410529 10.1038/nrm.2017.138

[10] M. Kinoshita, N. Matsumori, Inimitable impacts of ceramides on lipid rafts formed in artificial and natural cell membranes. Membranes 12, 727 (2022). 35893445 10.3390/membranes12080727 PMC9330320

[11] L. C. Silva, R. F. M. de Almeida, B. M. Castro, A. Fedorov, M. Prieto, Ceramide-domain formation and collapse in lipid rafts: Membrane reorganization by an apoptotic lipid. Biophys. J. 92, 502–516 (2007). 17056734 10.1529/biophysj.106.091876 PMC1751408

[12] L. J. Pike, Rafts defined: A report on the Keystone symposium on lipid rafts and cell function. J. Lipid Res. 47, 1597–1598 (2006). 16645198 10.1194/jlr.E600002-JLR200

[13] H. Heerklotz, Triton promotes domain formation in lipid raft mixtures. Biophys. J. 83, 2693–2701 (2002). 12414701 10.1016/S0006-3495(02)75278-8 PMC1302353

[14] M. Robinson, C. T. Filice, D. M. McRae, Z. Leonenko, Atomic force microscopy and other scanning probe microscopy methods to study nanoscale domains in model lipid membranes. Adv. Phys. X 8, 2197623 (2023).

[15] I. Levental, K. R. Levental, F. A. Heberle, Lipid rafts: Controversies resolved, mysteries remain. Trends Cell Biol. 30, 341–353 (2020). 32302547 10.1016/j.tcb.2020.01.009 PMC7798360

[16] K. G. N. Suzuki, A. Kusumi, Refinement of singer-nicolson fluid-mosaic model by microscopy imaging: Lipid rafts and actin-induced membrane compartmentalization. Biochim. Biophys. Acta Biomembr. 1865, 184093 (2023). 36423676 10.1016/j.bbamem.2022.184093

[17] M. Lorizate, O. Terrones, J. A. Nieto-Garai, I. Rojo-Bartolomé, D. Ciceri, O. Morana, J. Olazar-Intxausti, A. Arboleya, A. Martin, M. Szynkiewicz, M. Calleja-Felipe, J. Bernardino de la Serna, F. X. Contreras, Super-resolution microscopy using a bioorthogonal-based cholesterol probe provides unprecedented capabilities for imaging nanoscale lipid heterogeneity in living cells. Small Methods 5, e2100430 (2021). 34928061 10.1002/smtd.202100430

[18] H. Kemmoku, K. Takahashi, K. Mukai, T. Mori, K. M. Hirosawa, F. Kiku, Y. Uchida, Y. Kuchitsu, Y. Nishioka, M. Sawa, T. Kishimoto, K. Tanaka, Y. Yokota, H. Arai, K. G. N. Suzuki, T. Taguchi, Single-molecule localization microscopy reveals STING clustering at the trans-Golgi network through palmitoylation-dependent accumulation of cholesterol. Nat. Commun. 15, 220 (2024). 38212328 10.1038/s41467-023-44317-5 PMC10784591

[19] M. Maekawa, G. D. Fairn, Complementary probes reveal that phosphatidylserine is required for the proper transbilayer distribution of cholesterol. J. Cell Sci. 128, 1422–1433 (2015). 25663704 10.1242/jcs.164715

[20] J. D. Nickels, S. Chatterjee, C. B. Stanley, S. Qian, X. L. Cheng, D. A. A. Myles, R. F. Standaert, J. G. Elkins, J. Katsaras, The in vivo structure of biological membranes and evidence for lipid domains. PLOS Biol. 15, e2002214 (2017). 28542493 10.1371/journal.pbio.2002214 PMC5441578

[21] G. L. Nicolson, G. Ferreira de Mattos, The fluid–mosaic model of cell membranes: A brief introduction, historical features, some general principles, and its adaptation to current information. Biochim. Biophys. Acta Biomembr. 1865, 184135 (2023). 36746313 10.1016/j.bbamem.2023.184135

[22] A. J. Borst, Z. M. James, W. N. Zagotta, M. Ginsberg, F. A. Rey, F. DiMaio, M. Backovic, D. Veesler, The therapeutic antibody LM609 selectively inhibits ligand binding to human α_V_β_3_ integrin via steric hindrance. Structure 25, 1732–1739.e5 (2017). 29033288 10.1016/j.str.2017.09.007 PMC5689087

[23] J. Li, Y. Fukase, Y. Shang, W. Zou, J. M. Muñoz-Félix, L. Buitrago, J. van Agthoven, Y. Zhang, R. Hara, Y. Tanaka, R. Okamoto, T. Yasui, T. Nakahata, T. Imaeda, K. Aso, Y. Zhou, C. Locuson, D. Nesic, M. Duggan, J. Takagi, R. D. Vaughan, T. Walz, K. Hodivala-Dilke, S. L. Teitelbaum, M. A. Arnaout, M. Filizola, M. A. Foley, B. S. Coller, Novel pure α_V_β_3_ integrin antagonists that do not induce receptor extension, prime the receptor, or enhance angiogenesis at low concentrations. ACS Pharmacol. Transl. 2, 387–401 (2019). 10.1021/acsptsci.9b00041 PMC7088984 32259072

[24] C. Eggeling, A. Honigmann, Closing the gap: The approach of optical and computational microscopy to uncover biomembrane organization. Biochim. Biophys. Acta Biomembr. 1858, 2558–2568 (2016). 10.1016/j.bbamem.2016.03.025 27039279

[25] A. L. Duncan, W. Pezeshkian, Mesoscale simulations: An indispensable approach to understand biomembranes. Biophys. J. 122, 1883–1889 (2023). 36809878 10.1016/j.bpj.2023.02.017 PMC10257116

[26] J. Fan, M. Sammalkorpi, M. Haataja, Lipid microdomains: Structural correlations, fluctuations, and formation mechanisms. Phys. Rev. Lett. 104, 118101 (2010). 20366502 10.1103/PhysRevLett.104.118101

[27] J. Fan, M. Sammalkorpi, M. Haataja, Influence of nonequilibrium lipid transport, membrane compartmentalization, and membrane proteins on the lateral organization of the plasma membrane. Phys. Rev. E 81, 011908 (2010). 10.1103/PhysRevE.81.011908 20365400

[28] J. Fan, M. Sammalkorpi, M. Haataja, Formation and regulation of lipid microdomains in cell membranes: Theory, modeling, and speculation. FEBS Lett. 584, 1678–1684 (2010). 19854186 10.1016/j.febslet.2009.10.051

[29] M. Krieg, G. Fläschner, D. Alsteens, B. M. Gaub, W. H. Roos, G. J. L. Wuite, H. E. Gaub, C. Gerber, Y. F. Dufrêne, D. J. Müller, Atomic force microscopy-based mechanobiology. Nat. Rev. Phys. 1, 41–57 (2019).

[30] M. Li, N. Xi, L. Liu, Hierarchical micro-/nanotopography for tuning structures and mechanics of cells probed by atomic force microscopy. IEEE Trans. Nanobioscience 20, 543–553 (2021). 34242170 10.1109/TNB.2021.3096056

[31] L. Zhang, L. Zhao, P.-K. Ouyang, P. Chen, Insight into the role of cholesterol in modulation of morphology and mechanical properties of CHO-K1 cells: An in situ AFM study. Front. Chem. Sci. Eng. 13, 98–107 (2019).

[32] M. Shibata, H. Watanabe, T. Uchihashi, T. Ando, R. Yasuda, High-speed atomic force microscopy imaging of live mammalian cells. Biophys. Physicobiol. 14, 127–135 (2017). 28900590 10.2142/biophysico.14.0_127 PMC5590786

[33] Y. Shan, H. Wang, The structure and function of cell membranes examined by atomic force microscopy and single-molecule force spectroscopy. Chem. Soc. Rev. 44, 3617–3638 (2015). 25893228 10.1039/c4cs00508b

[34] J. Iturri, A. Weber, A. Moreno-Cencerrado, M. dM Vivanco, R. Benítez, S. Leporatti, J. L. Toca-Herrera, Resveratrol-induced temporal variation in the mechanical properties of MCF-7 breast cancer cells investigated by atomic force microscopy. Int. J. Mol. Sci. 20, 3275 (2019). 31277289 10.3390/ijms20133275 PMC6651212

[35] X. Wu, X. Yu, C. Chen, C. Chen, Y. Wang, D. Su, L. Zhu, Fibrinogen and tumors. Front. Oncologia 14, 1393599 (2024). 10.3389/fonc.2024.1393599 PMC11109443 38779081

[36] H.-Y. Lin, L. Lansing, J.-M. Merillon, F. B. Davis, H.-Y. Tang, A. Shih, X. Vitrac, S. Krisa, T. Keating, H. J. Cao, J. Bergh, S. Quackenbush, P. J. Davis, Integrin α_V_β_3_ contains a receptor site for resveratrol. FASEB J. 20, 1742–1744 (2006). 16790523 10.1096/fj.06-5743fje

[37] Y. Ho, Z. Li, Y. J. Shih, Y.-R. Chen, K. Wang, J. Whang-Peng, H.-Y. Lin, P. J. Davis, Integrin α_V_β_3_ in the mediating effects of dihydrotestosterone and resveratrol on breast cancer cell proliferation. Int. J. Mol. Sci. 21, 2906 (2020). 32326308 10.3390/ijms21082906 PMC7216104

[38] X. Pang, X. He, Z. Qiu, H. Zhang, R. Xie, Z. Liu, Y. Gu, N. Zhao, Q. Xiang, Y. Cui, Targeting integrin pathways: Mechanisms and advances in therapy. Signal. Transduct. Target. Ther. 8, 1 (2023). 36588107 10.1038/s41392-022-01259-6 PMC9805914

[39] T. M. Odrljin, C. G. Haidaris, N. B. Lerner, P. J. Simpson-Haidaris, Integrin α_V_β_3_-mediated endocytosis of immobilized fibrinogen by A549 lung alveolar epithelial cells. Am. J. Respir. Cell Mol. Biol. 24, 12–21 (2001). 11152645 10.1165/ajrcmb.24.1.3992

[40] V. Reyhani, P. Seddigh, B. Guss, R. Gustafsson, L. Rask, K. Rubin, Fibrin binds to collagen and provides a bridge for α_V_β_3_ integrin-dependent contraction of collagen gels. Biochem. J. 462, 113–123 (2014). 24840544 10.1042/BJ20140201 PMC4109839

[41] D. P. Ly, K. M. Zazzali, S. A. Corbett, De novo expression of the integrin α_5_β_1_ regulates αvβ3-mediated adhesion and migration on fibrinogen. J. Biol. Chem. 278, 21878–21885 (2003). 12676956 10.1074/jbc.M212538200

[42] M. M. Pesho, K. Bledzka, L. Michalec, C. S. Cierniewski, E. F. Plow, The specificity and function of the metal-binding sites in the integrin β_3_ A-domain. J. Biol. Chem. 281, 23034–23041 (2006). 16723352 10.1074/jbc.M602856200

[43] P. Zhang, T. Ozdemir, C.-Y. Chung, G. P. Robertson, C. Dong, Sequential binding of α_V_β_3_ and ICAM-1 determines fibrin-mediated melanoma capture and stable adhesion to CD11b/CD18 on neutrophils. J. Immunol. 186, 242–254 (2011). 21135163 10.4049/jimmunol.1000494 PMC3058329

[44] M. Rolli, E. Fransvea, J. Pilch, A. Saven, B. Felding-Habermann, Activated integrin α_V_β_3_ cooperates with metalloproteinase MMP-9 in regulating migration of metastatic breast cancer cells. Proc. Natl. Acad. Sci. U.S.A. 100, 9482–9487 (2003). 12874388 10.1073/pnas.1633689100 PMC170944

[45] P. J. Davis, S. A. Mousa, V. Cody, H.-Y. Tang, H.-Y. Lin, Small molecule hormone or hormone-like ligands of integrin α_V_β_3_: Implications for cancer cell behavior. Horm. Cancer 4, 335–342 (2013). 23943159 10.1007/s12672-013-0156-8 PMC10358123

[46] H. J. Garrigues, L. K. DeMaster, Y. E. Rubinchikova, T. M. Rose, KSHV attachment and entry are dependent on α_V_β_3_ integrin localized to specific cell surface microdomains and do not correlate with the presence of heparan sulfate. Virology. 464-465, 118–133 (2014). 25063885 10.1016/j.virol.2014.06.035 PMC4157101

[47] T. Gianni, V. Gatta, G. Campadelli-Fiume, α_V_β_3_-Integrin routes herpes simplex virus to an entry pathway dependent on cholesterol-rich lipid rafts and dynamin2. Proc. Natl. Acad. Sci. U.S.A. 107, 22260–22265 (2010). 21135248 10.1073/pnas.1014923108 PMC3009828

[48] S. Chakraborty, M. ValiyaVeettil, S. Sadagopan, N. Paudel, B. Chandran, c-Cbl-mediated selective virus-receptor translocations into lipid rafts regulate productive Kaposi’s sarcoma-associated herpesvirus infection in endothelial cells. J. Virol. 85, 12410–12430 (2011). 21937638 10.1128/JVI.05953-11 PMC3209366

[49] F. Orsini, A. Cremona, P. Arosio, P. A. Corsetto, G. Montorfano, A. Lascialfari, A. M. Rizzo, Atomic force microscopy imaging of lipid rafts of human breast cancer cells. Biochim. Biophys. Acta Biomembr. 1818, 2943–2949 (2012). 10.1016/j.bbamem.2012.07.024 22884468

[50] J. A. Peruzzi, T. F. Gunnels, H. I. Edelstein, P. Lu, D. Baker, J. N. Leonard, N. P. Kamat, Enhancing extracellular vesicle cargo loading and functional delivery by engineering protein-lipid interactions. Nat. Commun. 15, 5618 (2024). 38965227 10.1038/s41467-024-49678-z PMC11224323

[51] B. Kollmitzer, P. Heftberger, R. Podgornik, J. F. Nagle, G. Pabst, Bending rigidities and interdomain forces in membranes with coexisting lipid domains. Biophys. J. 108, 2833–2842 (2015). 26083923 10.1016/j.bpj.2015.05.003 PMC4472082

[52] Y. Tian, Y. Wu, L. Liu, L. He, J. Gao, L. Zhou, F. Yu, S. Yu, H. Wang, The structural characteristics of mononuclear-macrophage membrane observed by atomic force microscopy. J. Struct. Biol. 206, 314–321 (2019). 30946900 10.1016/j.jsb.2019.04.002

[53] C. Bernard, A. R. Carotenuto, N. M. Pugno, M. Fraldi, L. Deseri, Modelling lipid rafts formation through chemo-mechanical interplay triggered by receptor–ligand binding. Biomech. Model. Mechanobiol. 23, 485–505 (2024). 38060155 10.1007/s10237-023-01787-2 PMC10963483

[54] W. Chen, J. Lou, J. Hsin, K. Schulten, S. C. Harvey, C. Zhu, Molecular dynamics simulations of forced unbending of integrin α_V_β_3_. PLOS Comput. Biol. 7, e1001086 (2011). 21379327 10.1371/journal.pcbi.1001086 PMC3040657

[55] H. K. Gaikwad, S. V. Jaswandkar, K. S. Katti, A. Haage, D. R. Katti, Molecular basis of conformational changes and mechanics of integrins. Philos. Trans. R. Soc. A 381, 20220243 (2023). 10.1098/rsta.2022.0243 37211038

[56] Y. Chen, H. Lee, H. Tong, M. Schwartz, C. Zhu, Force regulated conformational change of integrin α_V_β_3_. Matrix Biol. 60-61, 70–85 (2017). 27423389 10.1016/j.matbio.2016.07.002 PMC5237428

[57] F. Lu, L. Zhu, T. Bromberger, J. Yang, Q. Yang, J. Liu, E. F. Plow, M. Moser, J. Qin, Mechanism of integrin activation by talin and its cooperation with kindlin. Nat. Commun. 13, 2362 (2022). 35488005 10.1038/s41467-022-30117-w PMC9054839

[58] J. Li, M. H. Jo, J. Yan, T. Hall, J. Lee, U. López-Sánchez, S. Yan, T. Ha, T. A. Springer, Ligand binding initiates single-molecule integrin conformational activation. Cell 187, 2990–3005.e17 (2024). 38772370 10.1016/j.cell.2024.04.049 PMC11162317

[59] O. Schussler, J. C. Chachques, M. Alifano, Y. Lecarpentier, Key roles of RGD-recognizing integrins during cardiac development, on cardiac cells, and after myocardial infarction. J. Cardiovasc. Transl. Res. 15, 179–203 (2022). 34342855 10.1007/s12265-021-10154-4

[60] C. Gest, U. Joimel, L. Huang, L.-L. Pritchard, A. Petit, C. Dulong, C. Buquet, C.-Q. Hu, P. Mirshahi, M. Laurent, F. Fauvel-Lafève, L. Cazin, J.-P. Vannier, H. Lu, J. Soria, H. Li, R. Varin, C. Soria, Rac3 induces a molecular pathway triggering breast cancer cell aggressiveness: Differences in MDA-MB-231 and MCF-7 breast cancer cell lines. BMC Cancer 13, 63 (2013). 23388133 10.1186/1471-2407-13-63 PMC3576359

[61] T. K. Harden, S. N. Hicks, J. Sondek, Phospholipase C isozymes as effectors of Ras superfamily GTPases. J. Lipid Res. 50, S243–S248 (2009). 19033212 10.1194/jlr.R800045-JLR200 PMC2674739

[62] J. Xu, X. Huang, Lipid metabolism at membrane contacts: Dynamics and functions beyond lipid homeostasis. Front. Cell Dev. Biol. 8, 615856 (2020). 33425923 10.3389/fcell.2020.615856 PMC7786193

[63] S. L. Veatch, P. Cicuta, P. Sengupta, A. Honerkamp-Smith, D. Holowka, B. Baird, Critical fluctuations in plasma membrane vesicles. ACS Chem. Biol. 3, 287–293 (2008). 18484709 10.1021/cb800012x

[64] J. Gómez, F. Sagués, R. Reigada, Nonequilibrium patterns in phase-separating ternary membranes. Phys. Rev. E 80, 011920 (2009). 10.1103/PhysRevE.80.011920 19658742

[65] J. Chen, Q. Zou, Large-range high-speed dynamic-mode atomic force microscope imaging: Adaptive tapping towards minimal force. Nanotechnology 34, 455503 (2023). 10.1088/1361-6528/acd700 37207634

[66] J. M. Green, A. Zheleznyak, J. Chung, F. P. Lindberg, M. Sarfati, W. A. Frazier, E. J. Brown, Role of cholesterol in formation and function of a signaling complex involving α_V_β_3_, integrin-associated protein (CD47), and heterotrimeric G proteins. J. Cell Biol. 146, 673–682 (1999). 10444074 10.1083/jcb.146.3.673 PMC2150554

[67] A. Cormier, M. G. Campbell, S. Ito, S. Wu, J. Lou, J. Marks, J. L. Baron, S. L. Nishimura, Y. Cheng, Cryo-EM structure of the α_V_β_8_ integrin reveals a mechanism for stabilizing integrin extension. Nat. Struct. Mol. Biol. 25, 698–704 (2018). 30061598 10.1038/s41594-018-0093-x PMC6214843

[68] X.-P. Xu, E. Kim, M. Swift, J. W. Smith, N. Volkmann, D. Hanein, Three-dimensional structures of full-length, membrane-embedded human α_IIb_β_3_ integrin complexes. Biophys. J. 110, 798–809 (2016). 26910421 10.1016/j.bpj.2016.01.016 PMC4776043

[69] R. I. Litvinov, M. Mravic, H. Zhu, J. W. Weisel, W. F. DeGrado, J. S. Bennett, Unique transmembrane domain interactions differentially modulate integrin α_V_β_3_ and α_IIb_β_3_ function. Proc. Natl. Acad. Sci. U.S.A. 116, 12295–12300 (2019). 31160446 10.1073/pnas.1904867116 PMC6589676

[70] Y. Chen, Z. Li, F. Kong, L. A. Ju, C. Zhu, Force-regulated spontaneous conformational changes of integrins α_5_β_1_ and α_V_β_3_. ACS Nano 18, 299–313 (2024). 38105535 10.1021/acsnano.3c06253 PMC10786158

[71] I. Johnston, L. J. Johnston, Ceramide promotes restructuring of model raft membranes. Langmuir 22, 11284–11289 (2006). 17154617 10.1021/la061636s

[72] S. Chiantia, N. Kahya, J. Ries, P. Schwille, Effects of ceramide on liquid-ordered domains investigated by simultaneous AFM and FCS. Biophys. J. 90, 4500–4508 (2006). 16565041 10.1529/biophysj.106.081026 PMC1471841

