# Supplementary material for: Visualizing dynamics of membrane rafts on live cells
Source: Sci Adv. 2025 Nov 28;11(48):eadv7001. doi: 10.1126/sciadv.adv7001 (PMC12662213; doi:10.1126/sciadv.adv7001)
Supplement: Supplementary file 1 — Supplementary Text Figs. S1 to S14 Data S1 Legends for movies S1 to S5 Tables S1 and S2 References [file sciadv.adv7001_sm.pdf]

Science Advances
AAAS

Supplementary Materials for

Visualizing dynamics of membrane rafts on live cells

Hsiang-Ling Chuang *et al.*

Corresponding author: Richard P. Cheng, rpcheng@ntu.edu.tw; Li-Chen Wu, lw25@ncnu.edu.tw; Ja-an Annie Ho, jaho@ntu.edu.tw; Chun-hsien Chen, chhchen@ntu.edu.tw

Sci. Adv. **11**, eadv7001 (2025)
DOI: 10.1126/sciadv.adv7001

The PDF file includes:

Supplementary Text
Figs. S1 to S14
Data S1
Legends for movies S1 to S5
Tables S1 and S2
References

Other Supplementary Material for this manuscript includes the following:

Movies S1 to S5

Supplementary Text

Basics of AFM scanning modes employed in this study

In this study, AFM was utilized as a label-free observation tool to identify the presence of membrane rafts on the surface of live MCF-7 cells. Two types of scanners and scanning modes were applied to obtain the physical properties of cell membrane. The measurements were carried out by (1) PeakForce® Quantitative Nanomechanics (QNM) mode AFM equipped with an Icon scanner and (2) Tapping Mode™ AFM equipped with a FastScan scanner. The features of membrane rafts were firstly interpreted with higher and stiffer than their surroundings in PeakForce® QNM mode images. Such information conduces the recognition of membrane rafts by Tapping Mode AFM. Specifically, phase angles measured by phase mode indicate different degrees of surface hardness which can be considered as liquid-ordered or liquid-disordered phase.

PeakForce® Quantitative Nanomechanics (QNM) mode. Peak force represents the maximum repulsion between the tip and the surface of the sample. For the same composition, the greater the repulsion, the higher the sample. To the AFM tip applied are a constant force and a frequency far below the cantilever resonant frequency. The tip taps on the sample surface with a lifted height and a frequency of 2000 times/sec by the Z piezo, corresponding to an intermittent and short contact time of 0.5 msec. For soft samples, it is beneficial to operate under the maximum constant force and utilize the real-time force as the linear feedback control. Images by modes of PeakForce Tapping, PeakForce Error, and PeakForce QNM are typically acquired simultaneously.

The signal of PeakForce Tapping is in distance (*i.e.*, in height, nm) and comes from the voltage output to the Z piezo which is calculated by the feedback control of the Digital Signal Processor (DSP). The images of PeakForce Error present the difference between the pre-set value of peak force (in Newton) and the measured one during the imaging.

In this study, the hardness of cellular membranes was evaluated by energy dissipation measured by PeakForce® QNM. Dissipation manifested the energy loss of the tip mechanical motion upon interacting with samples. To quantify the dissipation upon tip-sample interactions, the force curves of the tip approaching to and retracting from the sample were measured first. The amount of energy loss was derived from the difference in the integral area of the two force curves. A smaller (stronger) level of energy dissipation indicated a harder (softer) region and thus weaker (stronger) tip-sample interactions.

Tapping Mode™. In this scanning mode, the AFM tip is operating under the resonant frequency of the cantilever. Simultaneously obtained images include modes of (1) height, (2) amplitude error, (3) phase, and (4) TM (Tapping Mode) deflection. For the height mode, the imaging procedures are identical to those of PeakForce Tapping mode. The image of amplitude error produces a map of the error between the setpoint value and the actual amplitude of the tip as it passes through the monitored positions of the sample. The error would be negative (positive) while the tip pass through elevated (lowered) height. Phase mode shows the phase difference between the frequency that drives the cantilever oscillation and the frequency after the tip-sample interactions by monitoring the laser beam reflected from the tip. The image of phase difference, either the lag or lead of the tip relative to that of the oscillation-driving piezo, reveals the distribution of softer or harder sample regions although the assignment is not as clear as that of PeakForce QNM mode. TM deflection mode measures the extent of cantilever deflection signal (in distance or volts) to which an additional feedback loop is introduced to regulate the mean value of the tip vibration such that the sample topography can be better tracked and enhanced (*65*). The higher extent of deflection, the higher elevation of sample.

Hadamard product-treated AFM images of live MCF-7 cells

The operation of Hadamard product, for two matrices A and B with the same dimension, results in a matrix with elements $A_{ij} \times B_{ij} = C_{ij}$. To reduce the complexity of raw AFM images of live MCF-7 cells in this study, elements in matrices A and B were pixels in images corresponding to height and hardness. The two parameters were employed to describe the membrane rafts based on the following considerations. *The 2006 Keystone Symposium on Lipid Rafts and Cell Function* reached a consensus which defined membrane rafts by "small (10−200 nm), heterogeneous, highly dynamic, sterol- and sphingolipid-enriched domains" (*12*). The "sterol- and sphingolipid-enriched domains" are presumably harder and taller than the surrounding lipid membranes. Additionally, our experimental design included the introduction of ligands for integrin $\alpha_V\beta_3$ which resided in membrane rafts (*36, 66*). After being activated by the ligands, the conformation of $\alpha_V\beta_3$ changed from a bent-close configuration to a fully extended structure (*23, 54-56, 67, 68*), causing an increase in height. Accordingly, parameters to be treated by the Hadamard product are height and hardness.

In this study live MCF-7 cells were imaged by PeakForce QNM® Mode (fig. S1A-C) and Tapping Mode (Fig. 2 in the main text). The PeakForce QNM® Mode provided the hardness information of the cell surface quantitatively to facilitate the assignment of membrane rafts by the Tapping Mode. Imaging was performed by PeakForce Tapping, PeakForce Error, and PeakForce QNM modes simultaneously. The first two modes revealed the morphological details of the cellular surface. It was straightforward to correlate the morphology with images obtained by the PeakForce Tapping Mode (in height, nanometer, fig. S1A), yet the PeakForce Error Mode (in force, Newton, fig. S1B) offered better spatial resolution. Images obtained by PeakForce QNM (fig. S1C) displayed energy dissipation, in which the energy loss of the tip mechanical motion upon interacting with the substrate corresponded to the substrate hardness. A lower (higher) level of energy dissipation indicated a harder (softer) region and was coded in red (blue). More detailed information of these modes is described in the previous section (Section 1 of this Supplementary Information).

Features that appear higher in row a of fig. S1 and also harder in row c are considered membrane rafts. Images in the third column (fig. S1, panels iii) are successively zoomed from the red squares indicated in the previous columns. The images in panels iv are those in panels iii but plotted in grayscale and mapped with grid lines to facilitate cross-examination. To pinpoint membrane rafts, the Hadamard product image (fig. S1E) was generated for panels $1A_{iv}$ and $1C_{iv}$ to screen out the soft or shallow features. The yellow circles mark examples where the features are higher but softer, or harder but not elevated from their surroundings. The red circles mark examples that are both higher and harder (*i.e.*, membrane rafts). Although cytoskeletons are also higher and stiffer, their elongated shapes can be readily differentiated.

Morphological analysis of protruded features: size, height, and persistent time

The main text addressed that, in a representative experiment, about 28% protruded nanodomains did not respond to the addition of Mn^{2+}/resveratrol. Hence, data of these non-responsive protrusions should be excluded from the discussion of ligand-activated persistent time. fig. S6B and S7C (uppermost panel) display, respectively, the height and persistent time distribution of the protrusions measured without the presence of Mn^{2+}/resveratrol. Based on the null hypothesis, ligand-activated protrusions were taller than 2.95 nm which was the upper boundary of the 95% confidence interval of fig. S6B. Accordingly, fig. S7A was prepared from the height evolution of protrusions growing taller than 2.95 nm. The histogram was fitted by Gaussian curves which

resolved distributions peaked at 3.28 (±0.19) nm, 4.67 (±0.97) nm, 7.01 (±0.97) nm, and 9.56 (±0.97) nm (fig. S7A). Those counts near the cut-off at 2.95 nm were very sharp (pink curve) and were regarded as fluctuation-caused background noise. The other three peaks were assumed to be different activated conformations. The monitoring of persistent time started (ended) when the protrusions taller than (levelled to) 3.70 nm, the lower boundary of the 95% confidence interval of the 4.67 (±0.97) nm peak (the green curve). The persistent time is defined as the duration from when the height exceeded 3.70 nm until it decreased back to 3.70 nm (fig. S7).

Integrin activation by Mn^{2+} for apoptosis induction in MCF-7 cells by resveratrol

For resveratrol-induced apoptosis of MCF-7 cells, fig. S8 manifests the necessity of integrin activation. Images in panels A and B were acquired in the medium, respectively, without and with the presence of Mn^{2+}, known to enable the activation of $\alpha_V\beta_3$ (*69, 70*). The event of cellular apoptosis was recorded by a confocal laser scanning microscope (Zeiss LSM 780) using Alexa Fluor® 488-conjugated Annexin V (green), a protein with high binding affinity to phosphatidylserine (PS). The phospholipid compositions at the outer and inner membranes are different. PS translocation from the inner side of the membrane to the outer leaflet is an indication of the early stage of apoptosis, commonly detected by the binding of PS exposed at the outer leaflet with dye-tagged Annexin V.

Resveratrol alone did not cause abnormal cell morphology under bright field microscopy (fig. S8A). The combined treatment with Mn^{2+} (50 μM)/resveratrol (10 μM) induced changes in the cells, including shrinkage and membrane blebbing (fig. S8A). The fluorescent intensity became apparent at 30 min and reached maximal at 60 min after drug treatment. The decrease in fluorescent intensity at > 90 min was attributed to photobleaching.

Confocal Imaging of Membrane Order and Activated Integrin $\alpha_V\beta_3$ Co-localization in MCF-7 Cells

MCF-7 cells were maintained in Dulbecco's Modified Eagle Medium (DMEM, Gibco) supplemented with 10% fetal bovine serum (FBS, Gibco) and 1% penicillin–streptomycin (Gibco) at 37 °C in a humidified incubator with 5% CO_2. For confocal microscopic imaging, 1×10^4 cells were seeded onto 35 mm glass-bottom confocal dishes (ibidi μ-Dish) and incubated overnight.

Cells were fixed with 10% neutral buffered formalin (Sigma-Aldrich) at room temperature for 10 min, rinsed twice with phosphate-buffered saline (PBS). Non-specific binding was blocked with 5% bovine serum albumin (BSA, Folibio) in PBS overnight at 4 °C. After two PBS washes, samples were stained with 2 μM C-Laurdan (MedChemExpress) at 37 °C for 20 min in the dark to visualize lipid ordered/disordered phases through imaging (excitation 405 nm; emission 430–460 nm and 480–510 nm).

Following initial C-Laurdan imaging, cells were permeabilized with 0.1% Triton X-100 (Sigma-Aldrich) in PBS for 10 min at room temperature, rinsed twice with PBS, and subjected to immunocytochemistry (ICC) staining. Samples were incubated overnight at 4 °C with mouse monoclonal anti-integrin α_V/β_3 antibody (Santa Cruz Biotechnology, Cat# sc-7312, 1:200 dilution), followed by two PBS washes and a 1 h incubation at room temperature in the dark with Alexa Fluor® 555-conjugate anti-mouse IgG (H+L), $F(ab')_2$ fragment secondary antibody (Cell Signaling Technology, Cat# 4409, 1:500 dilution).

All imaging were performed using a Zeiss LSM780 confocal microscope. Generalized polarization (GP) analysis of the C-Laurdan signal revealed lipid liquid-ordered (L_o) domains that exhibited strong spatial overlap with $\alpha_V\beta_3$ staining (fig. S10), indicating preferential localization of $\alpha_V\beta_3$ within raft-like, ordered membrane regions.

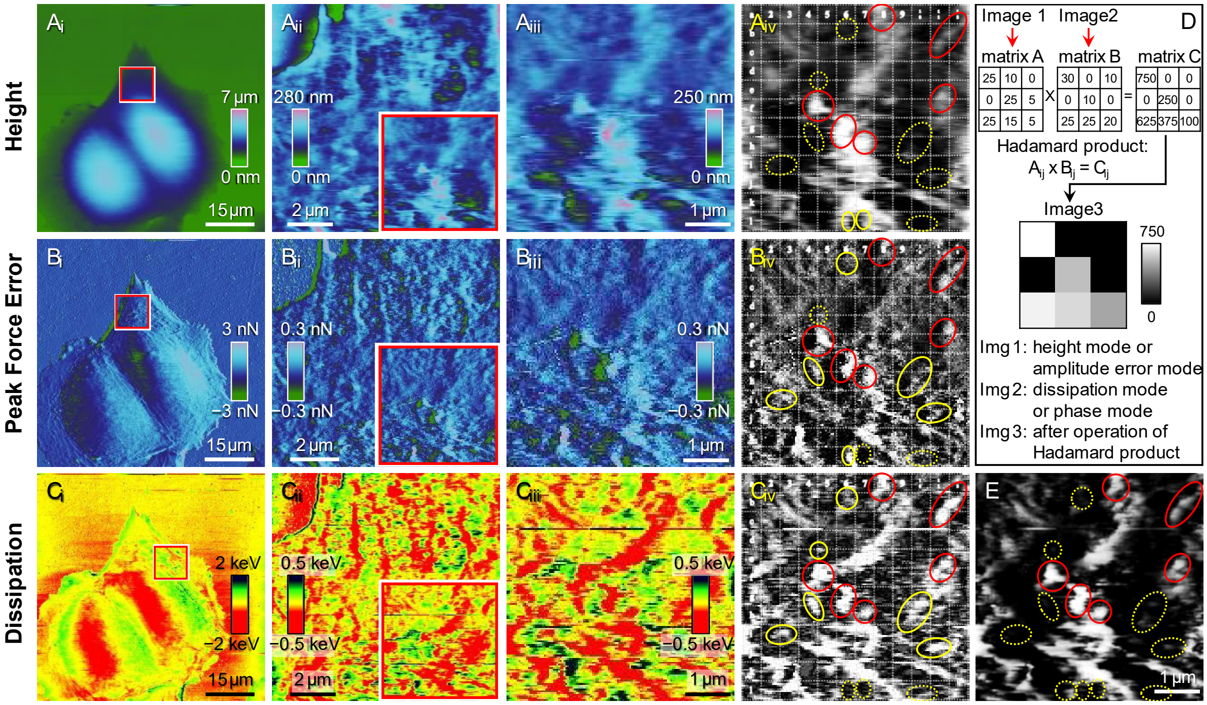

Fig. S1. Raw images of live MCF-7 cells by PeakForce QNM® Mode and Hadamard product image. Operation of Hadamard product manifests features higher and harder than their surroundings. PeakForce® QNM images obtained simultaneously by modes of (**A**) PeakForce Tapping, (**B**) PeakForce Error, and (**C**) PeakForce QNM. The boxes in panels i and ii indicate where panels ii and iii were successively zoomed and acquired from panels i. The grayscale images of panels iii are presented in panels iv where white (black) color symbolizes high (low) and hard (soft) regions. Red circles exemplify high and hard features while the yellow ones are either high but soft or hard but low. (**D**) Operation scheme of Hadamard product. (**E**) Image after the operation of Hadamard product for raw data of panels A_{iv} and C_{iv}. Image size: (A–C) i, 70 µm × 70 µm, ii, 10 µm × 10 µm, iii, 5 µm × 5 µm, iv, 5 µm × 5 µm; (E) 5 µm × 5 µm.

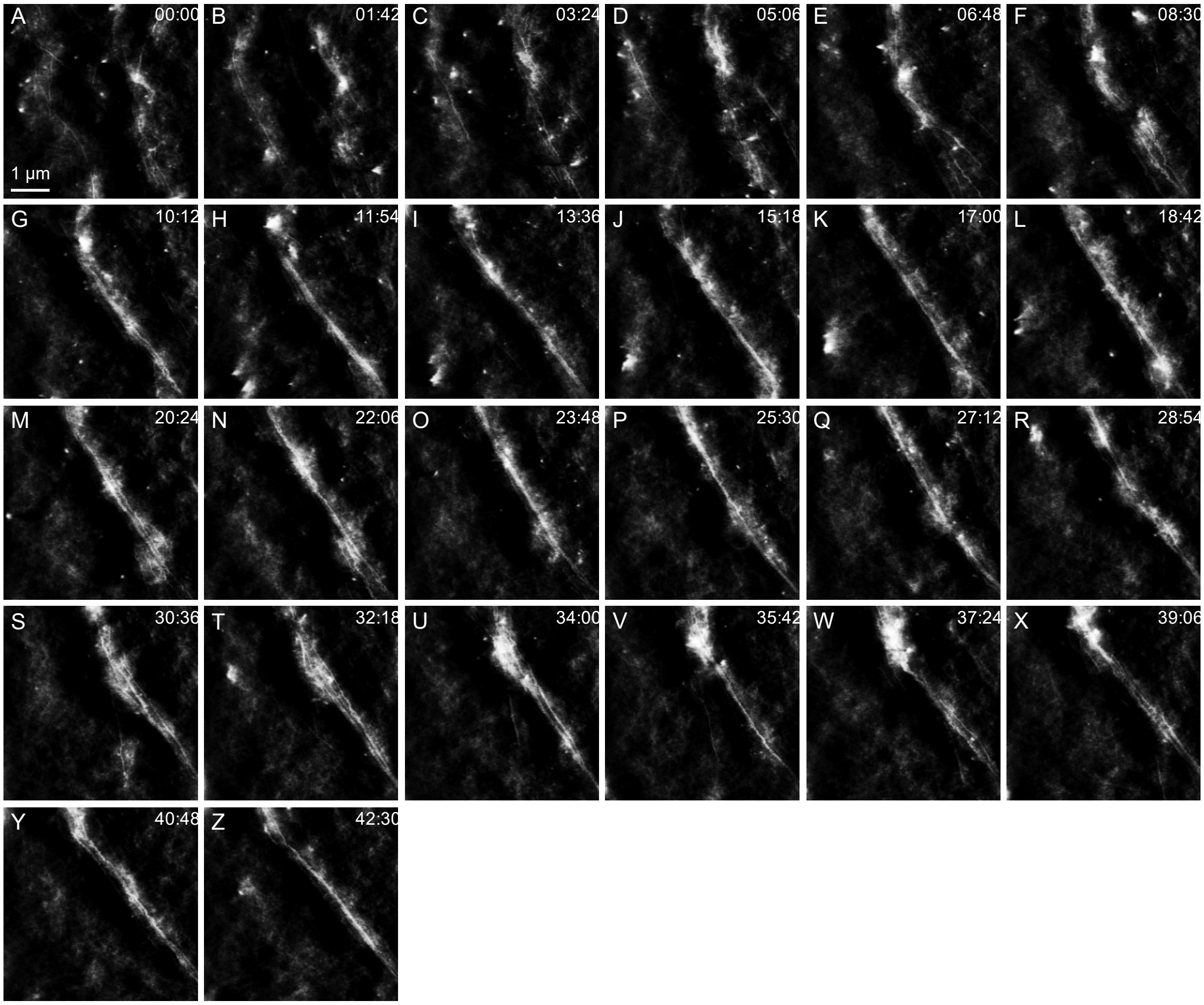

Fig. S2. AFM images to manifest the ease in distinguishing membrane rafts from cellular skeletons. The operation of Hadamard product for images of Amplitude Error Mode (e.g., Fig. $2B_{ii}$ in the main text) and Phase Mode (e.g., Fig. $2B_{iii}$) emphasizes higher and harder features that fit the description of membrane rafts and cellular skeletons. Their shapes and sizes are very different. Elongated and circular features are ascribed to the characteristics of cellular skeletons and membrane rafts, respectively. Image A is presented as panel D of Fig. 2 in the main text. Only images with the same tip scanning direction (from top to bottom) are displayed. Conditions: solution, blank PBS buffer (10 mM phosphate, pH 7.4); scan rate: 51 sec/frame; drive frequency: 110–130 kHz; image size: 5 μm × 5 μm.

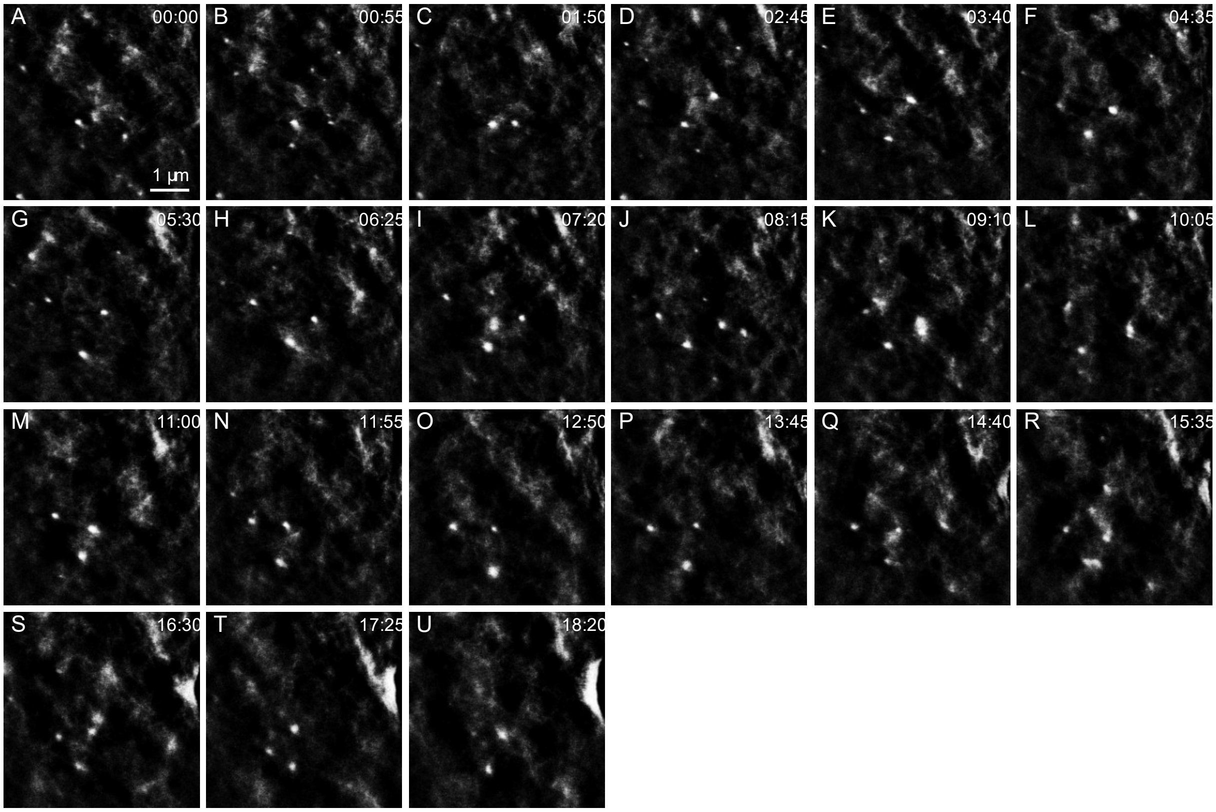

Fig. S3. Dynamics of membrane rafts on a live MCF-7 cell in plain phosphate buffer. Images are subject to the operation of Hadamard product as described in fig. S2. Images A–E are presented as panels A_i–A_v of Fig. 3 in the main text. Supplementary Movie S2 is prepared from this image set. Distinct from those in figs. S1 and S2 are that images of figs. S3–S5 and Supplementary Movies S1–S4 were intentionally performed on areas without major cellular skeletons in the image area. Conditions: solution, blank PBS buffer; scan rate: 28 sec/frame; image size: 5 μm × 5 μm. Other conditions were the same as fig. S2.

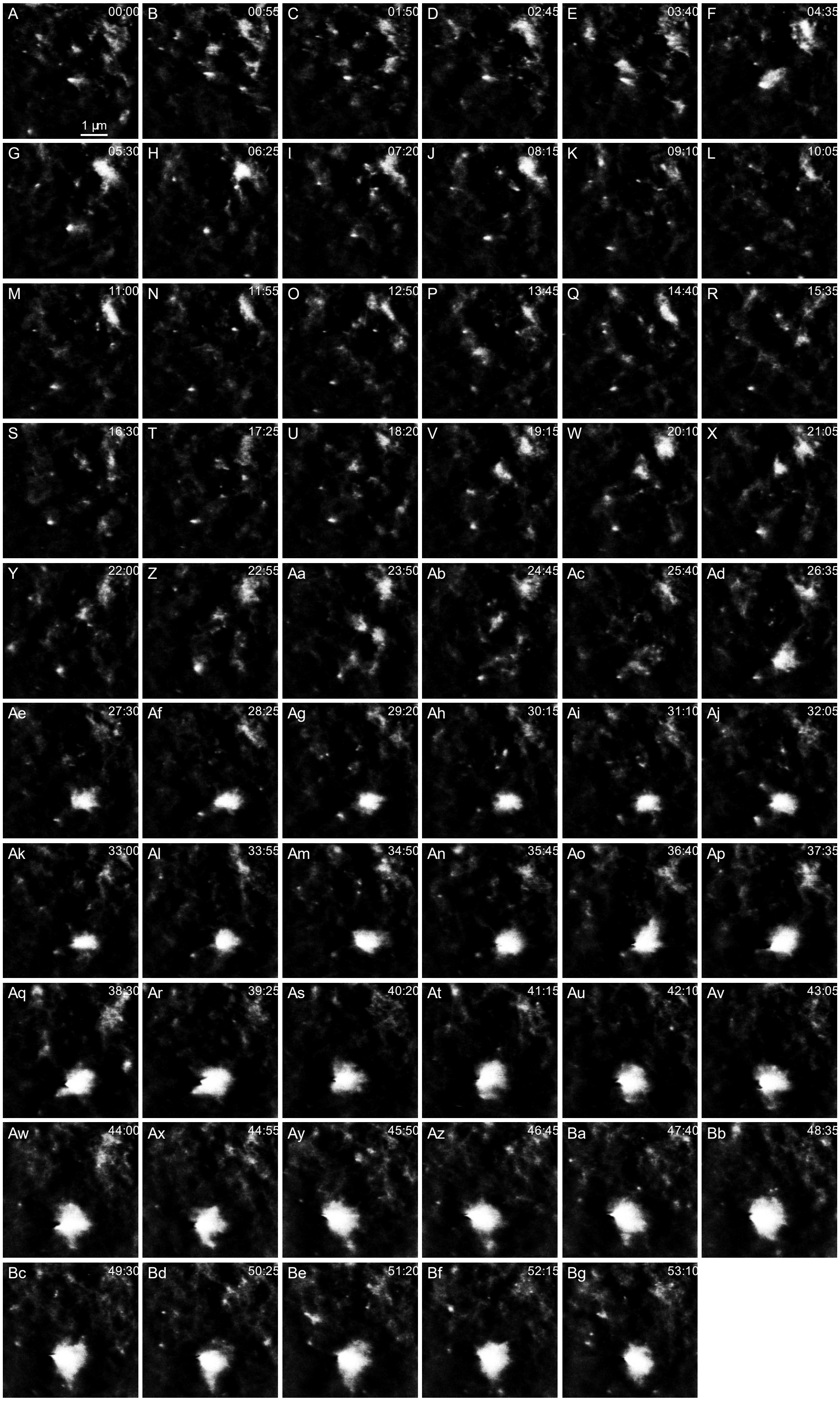

A 00:00
1 µm
B 00:55
C 01:50
D 02:45
E 03:40
F 04:35
G 05:30
H 06:25
I 07:20
J 08:15
K 09:10
L 10:05
M 11:00
N 11:55
O 12:50
P 13:45
Q 14:40
R 15:35
S 16:30
T 17:25
U 18:20
V 19:15
W 20:10
X 21:05
Y 22:00
Z 22:55
Aa 23:50
Ab 24:45
Ac 25:40
Ad 26:35
Ae 27:30
Af 28:25
Ag 29:20
Ah 30:15
Ai 31:10
Aj 32:05
Ak 33:00
Al 33:55
Am 34:50
An 35:45
Ao 36:40
Ap 37:35
Aq 38:30
Ar 39:25
As 40:20
At 41:15
Au 42:10
Av 43:05
Aw 44:00
Ax 44:55
Ay 45:50
Az 46:45
Ba 47:40
Bb 48:35
Bc 49:30
Bd 50:25
Be 51:20
Bf 52:15
Bg 53:10

Fig. S4. Dynamic response of membrane rafts to fibrinogen on a live MCF-7 cell. Images are subject to the operation of Hadamard product as described in fig. S1. There are coagulated entities with ~1-μm in size after administering of fibrinogen for ~5 min (in panel G) and ~26 min (in panel Ad). Images Z, Ab, Ac, Ad, and Af are presented, respectively, as panels B_i–B_v of Fig. 3 in the main text. Supplementary Movie S3 is prepared from this image set. Conditions: solution, PBS with 70 μM fibrinogen; scan rate: 28 sec/frame; image size: 5 μm × 5 μm. Other conditions were the same as fig. S2.

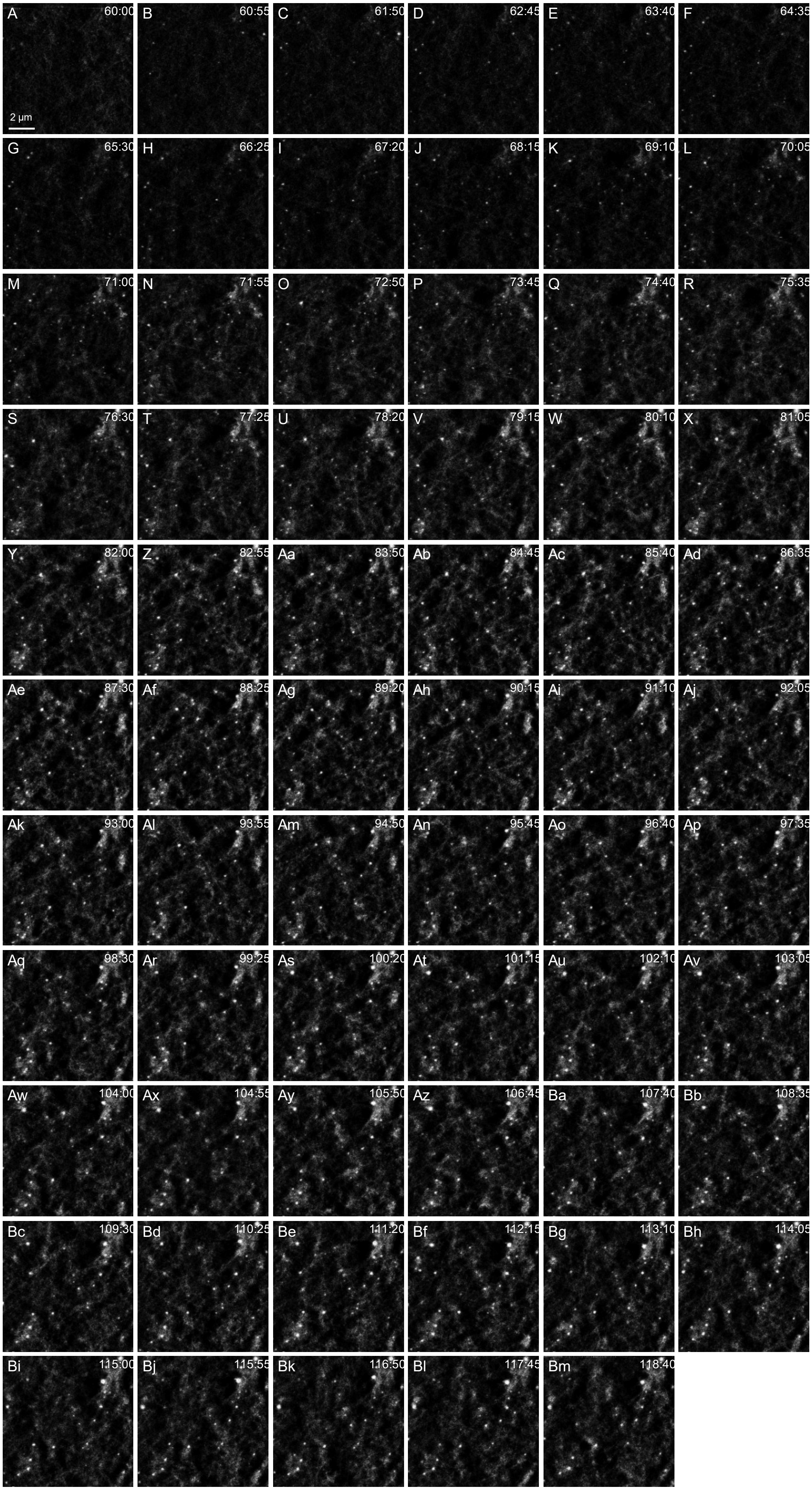
A 60:00
2 µm
B 60:55
C 61:50
D 62:45
E 63:40
F 64:35
G 65:30
H 66:25
I 67:20
J 68:15
K 69:10
L 70:05
M 71:00
N 71:55
O 72:50
P 73:45
Q 74:40
R 75:35
S 76:30
T 77:25
U 78:20
V 79:15
W 80:10
X 81:05
Y 82:00
Z 82:55
Aa 83:50
Ab 84:45
Ac 85:40
Ad 86:35
Ae 87:30
Af 88:25
Ag 89:20
Ah 90:15
Ai 91:10
Aj 92:05
Ak 93:00
Al 93:55
Am 94:50
An 95:45
Ao 96:40
Ap 97:35
Aq 98:30
Ar 99:25
As 100:20
At 101:15
Au 102:10
Av 103:05
Aw 104:00
Ax 104:55
Ay 105:50
Az 106:45
Ba 107:40
Bb 108:35
Bc 109:30
Bd 110:25
Be 111:20
Bf 112:15
Bg 113:10
Bh 114:05
Bi 115:00
Bj 115:55
Bk 116:50
Bl 117:45
Bm 118:40

Fig. S5. Dynamic response of membrane rafts to resveratrol on a live MCF-7 cell. Images are subject to the operation of Hadamard product as described in fig. S1. Presented images were acquired 60 min after introducing Mn^{2+}/resveratrol because it took ~1 hr for Mn^{2+} to activate integrin $\alpha_V\beta_3$ toward resveratrol (please see fig. S8). The density and heights (~15 nm) of the white protrusions are larger than those with plain PBS buffer (figs. S2 and S3). The white features clustered and formed plateaus in the upper right and lower left, yet individual protrusions did not fuse as those ~1-μm ones in the case of fibrinogen (fig. S4). Images K, Q, W, An, and As are presented, respectively, as panels C_i–C_v of Fig. 3 in the main text. Supplementary Movie S4 is prepared from this image set. Conditions: solution, PBS with 50 μM Mn^{2+} and 10 μM resveratrol; scan rate: 28 sec/frame; image size: 10 μm × 10 μm. Other conditions are the same as fig. S2.

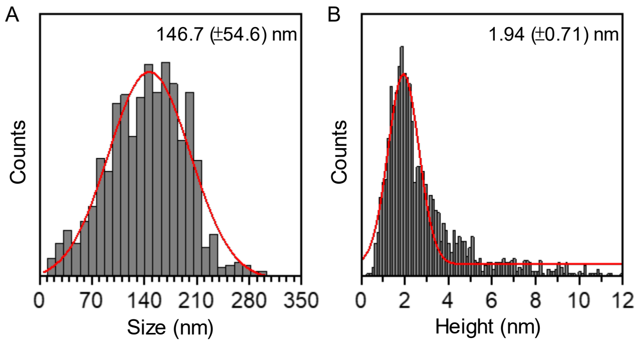

Fig. S6. Size and height distribution of protruded features on MCF-7 live cells. Histograms of (**A**) size and (**B**) height of features taller and harder than their surroundings. The experimental conditions were in PBS buffer without stimulants, the same as that described in Fig. 2 of the main text. The size and height were measured from Hadamard Product images and TM deflection images, respectively. The data were obtained from more than a thousand of protruded nanodomains. The red curves were fitted by Gaussian distributions and were peaked at 146.7 (±54.6) nm in size and 1.94 (±0.71) nm in height. Bin size: (A) 10.8 nm, (B) 0.12 nm.

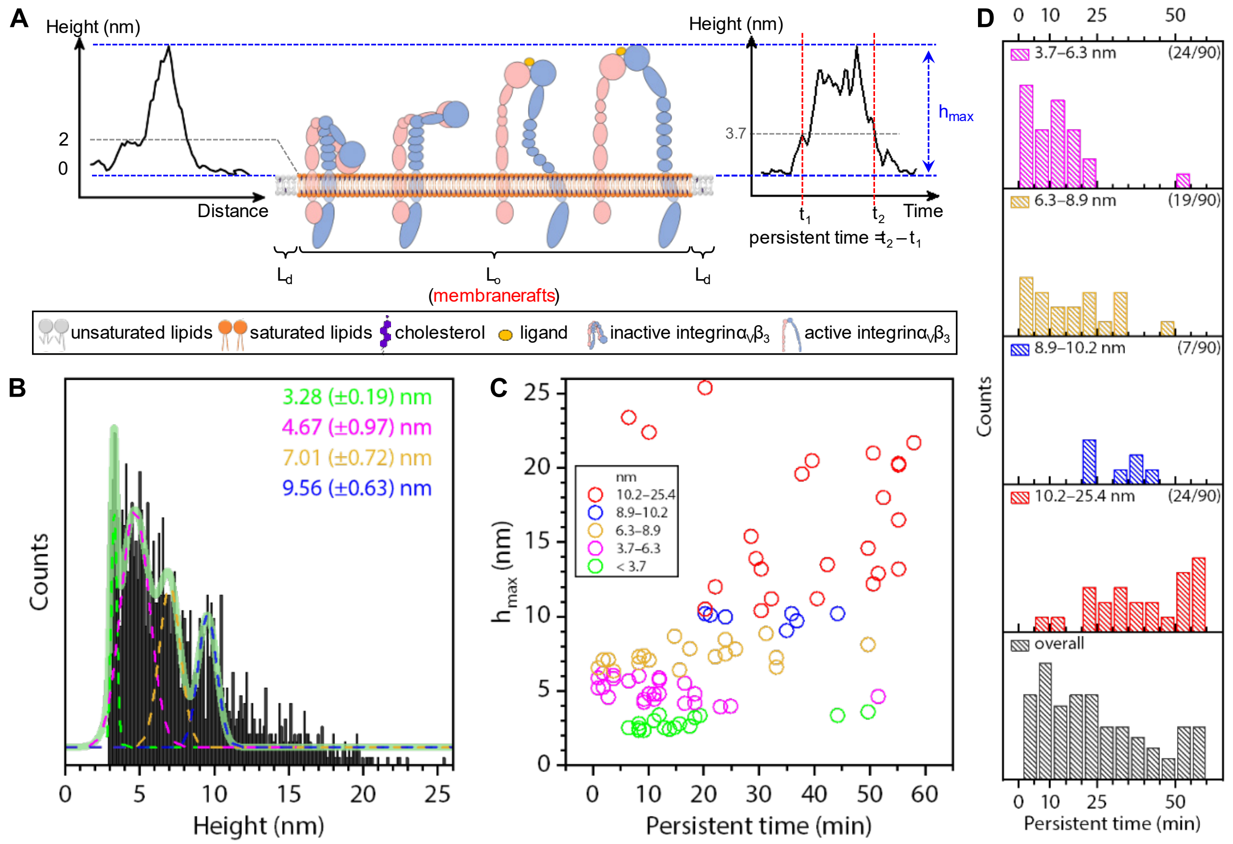

Fig. S7. Height and persistent time of Mn^{2+}/resveratrol-responsive protrusions. (**A**) Illustration to manifest AFM information about (left) section profile of a membrane raft and (right) height-time trace of a protrusion, (**B**) height histograms, (**C**) correlation of h_{max} with the persistent time, and (**D**) height-dependent persistent time for Mn^{2+}/resveratrol-responsive protrusions on an MCF-7 live cell. The information of protrusion height and persistent time was obtained from more than a thousand features from TM deflection images in fig. S5. Panel B did not incorporate data below 2.95 nm because those were within the 95% confidence interval of protrusion heights without being exposed to Mn^{2+}/resveratrol. The curves are fitted by Gaussian distributions and are peaked at 3.28 (±0.19) nm, 4.67 (±0.97) nm, 7.01 (±0.97) nm and 9.56 (±0.97) nm in height. The persistent time of an activated event was initiated upon the height becoming higher than 3.7 nm, based on the peak of the green curve. Explicitly, persistent time is defined as the duration during which the height remains above 3.7 nm. In the image set of fig. S5, there were 65 responsive events among 90 trackable membrane rafts, which were grouped in color based on the curves fit in panel B. The green (< 3.7 nm) and red (> 10 nm) groups may be interpreted as nonresponsive nanodomains and those with fully extended integrins, respectively. The biological meanings for the other two groups are elusive at this point. Entries for the 90 trackable nanodomains can be found at Data S1.

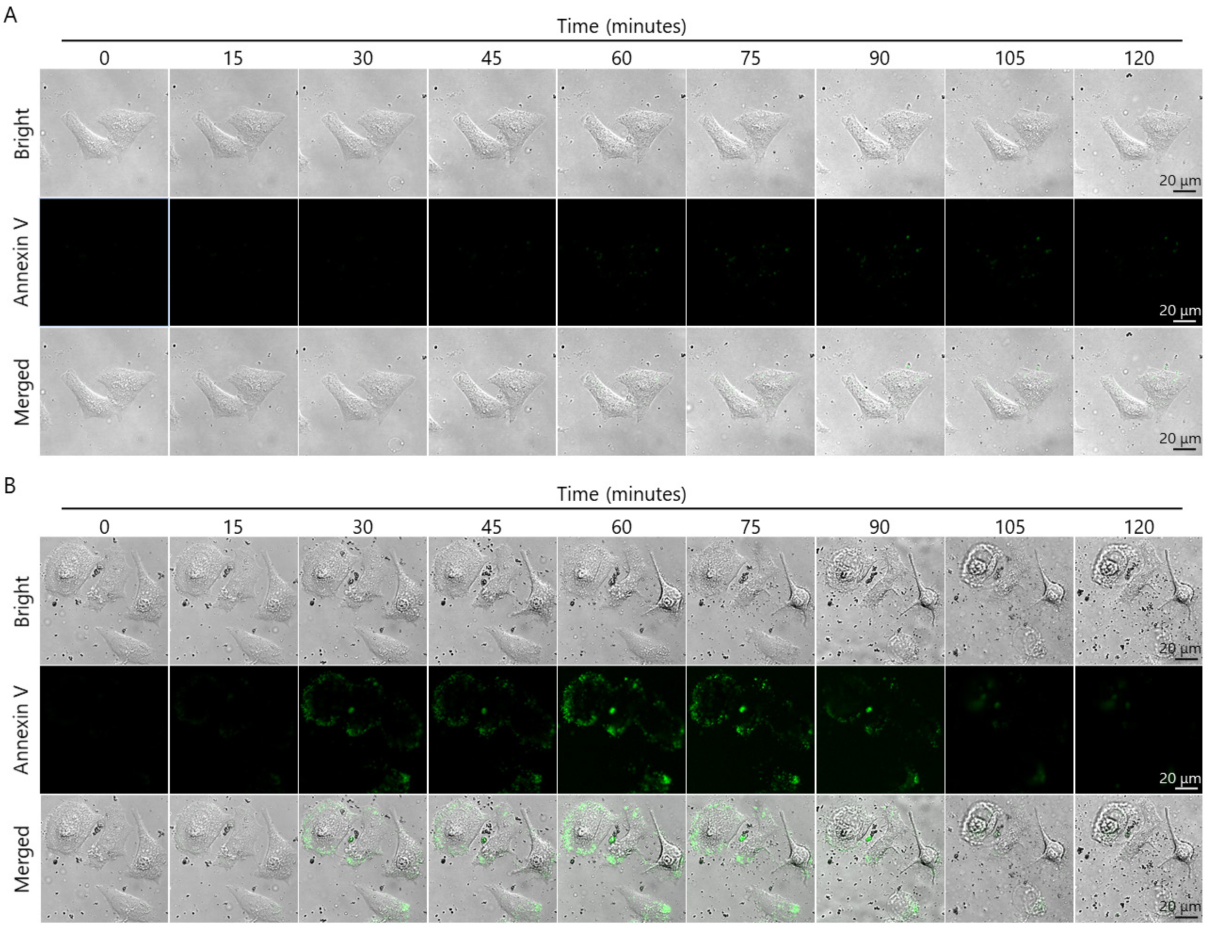

Fig. S8. Confocal microscope images indicating Mn^{2+}-activated integrin and resveratrol-induced cellular apoptosis. (**A**) 10 µM resveratrol only and (**B**) 50 µM Mn^{2+} and 10µM resveratrol were administered to MCF-7 cells in PBS containing Alexa Fluor® 488-conjugated Annexin V (green). Images were captured every 15 min after drug treatment, continuing until 120 min. The experimental environments were maintained at 37 °C in a humidified atmosphere of 5% CO_2.

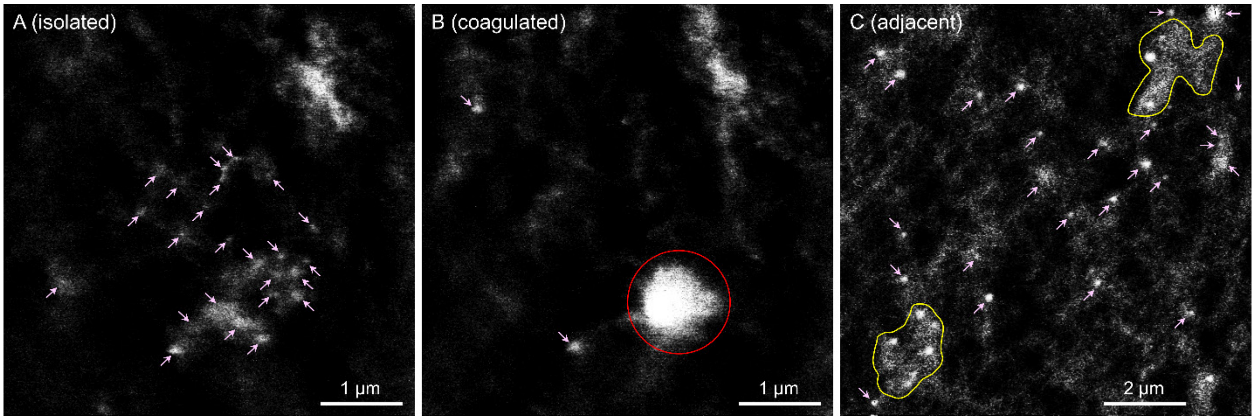

Fig. S9. AFM images to exemplify the "features" referred in footnote c of table S1. Pink arrows, red and yellow traces highlight the isolated feature, coagulated feature, and elevated terraces, respectively. Fig. S9A was obtained in PBS without the addition of stimulants. Fig. S9B and S9C were acquired after the MCF-7 cells were subjected to the treatment of fibrinogen and Mn^{2+}/resveratrol, respectively. Experimental conditions were the same as those for Fig. 3 in the main text. Image size: (A) 5 µm × 5 µm; (B) 5 µm × 5 µm; (C) 10 µm × 10 µm**.**

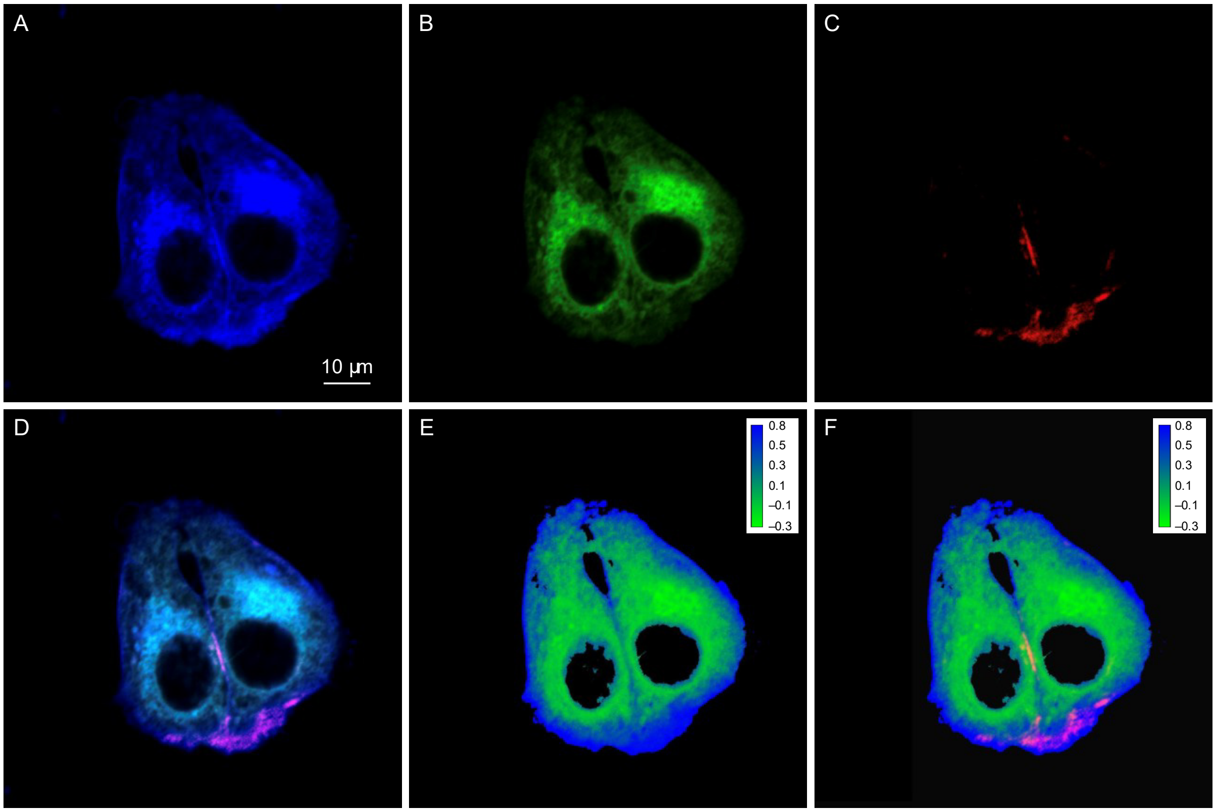

Fig. S10. Co-localization of integrin $\alpha_V\beta_3$ and lipid nanodomain structures on MCF-7 cells stained with C-Laurdan and visualized under confocal microscopy. (**A**,**B**) C-Laurdan emission from (L_o; excited at 405 nm, emission collected at 430–460 nm, blue) and liquid-disordered lipid nanodomain (L_d; excited at 405 nm, emission collected at 480–510 nm, green). (**C**) Immunohistochemical localization of integrin $\alpha_V\beta_3$ (red). (**D**) Merged image of integrin $\alpha_V\beta_3$, liquid-ordered lipid nanodomain, and liquid-disordered lipid nanodomain. (**E**) Generalized polarization (GP) image calculated using the equation GP = $(I_{440} - I_{490})/(I_{440} + I_{490})$, displayed with a customized LUT (range: –0.3 to 0.8; background masked). (**F**) Merge image showing co-localization of GP image and integrin $\alpha_V\beta_3$, confirming that $\alpha_V\beta_3$ is within liquid-ordered membrane domains (rafts). Scale bar: 10 μm.

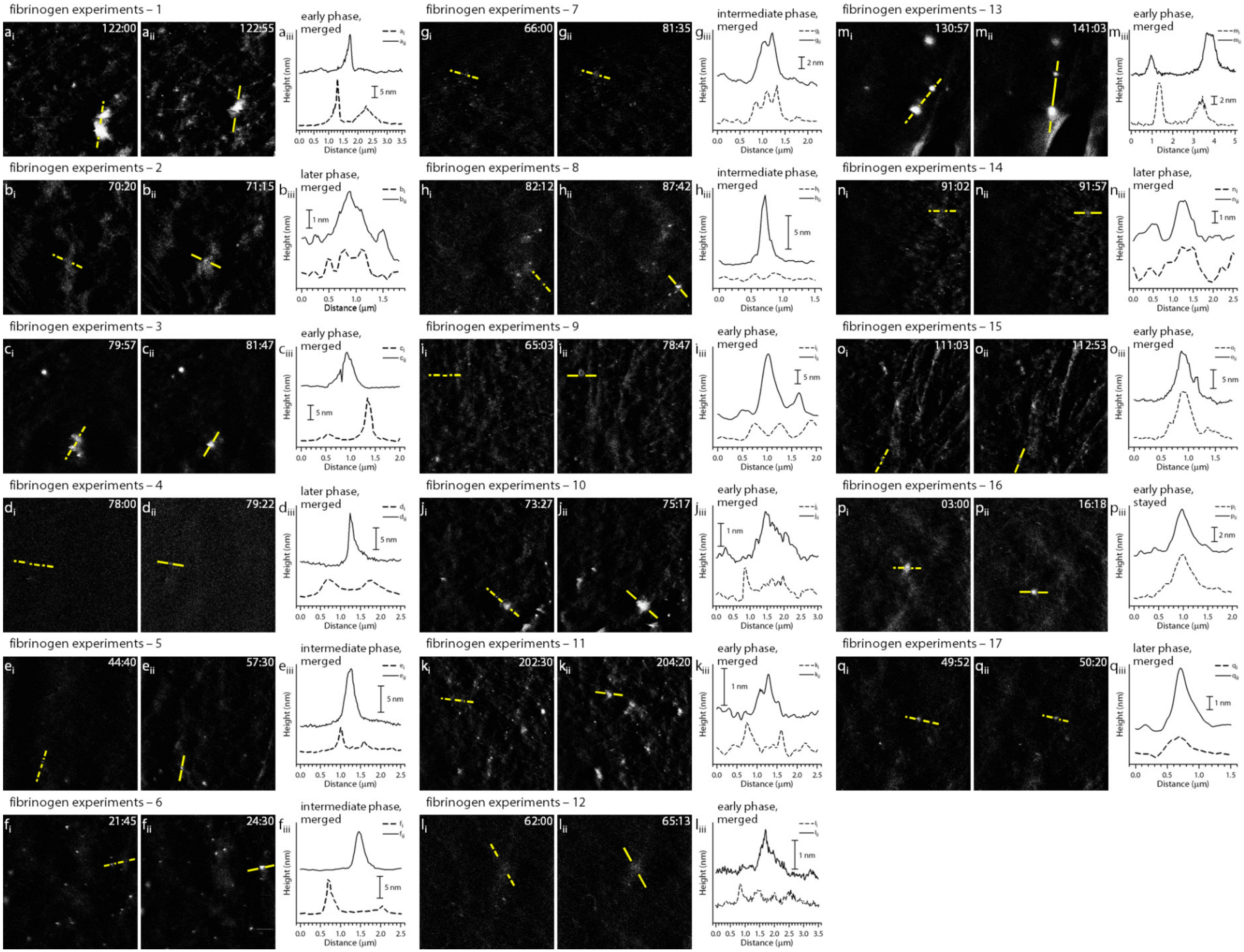
fibrinogen experiments – 1
122:00
122:55
early phase, merged
5 nm
fibrinogen experiments – 2
70:20
71:15
later phase, merged
1 nm
fibrinogen experiments – 3
79:57
81:47
early phase, merged
5 nm
fibrinogen experiments – 4
78:00
79:22
later phase, merged
5 nm
fibrinogen experiments – 5
44:40
57:30
intermediate phase, merged
5 nm
fibrinogen experiments – 6
21:45
24:30
intermediate phase, merged
5 nm
fibrinogen experiments – 7
66:00
81:35
intermediate phase, merged
2 nm
fibrinogen experiments – 8
82:12
87:42
intermediate phase, merged
5 nm
fibrinogen experiments – 9
65:03
78:47
early phase, merged
5 nm
fibrinogen experiments – 10
73:27
75:17
early phase, merged
1 nm
fibrinogen experiments – 11
202:30
204:20
early phase, merged
1 nm
fibrinogen experiments – 12
62:00
65:13
early phase, merged
1 nm
fibrinogen experiments – 13
130:57
141:03
early phase, merged
2 nm
fibrinogen experiments – 14
91:02
91:57
later phase, merged
1 nm
fibrinogen experiments – 15
111:03
112:53
early phase, merged
5 nm
fibrinogen experiments – 16
03:00
16:18
early phase, stayed
2 nm
fibrinogen experiments – 17
49:52
50:20
later phase, merged
1 nm
Height (nm)
Distance (μm)

Fig. S11. Representative AFM images of fibrinogen-activated MCF-7 cells. These images are from extensive experiments to support the finding reported in the main text. Due to the large volume of data, only two representative images per cell are displayed. In the 5-μm imaging zones on each cell, the responses described in the main text were observed in 18 out of 22 independent experiments−17 examples are shown here, with the remaining one presented in the main text. The displayed features include marked increases in protrusion size during early, intermediate, and later phase. The images were acquired by Tapping Mode and are processed by the operation of Hadamard product. Image size: 5 μm × 5 μm.

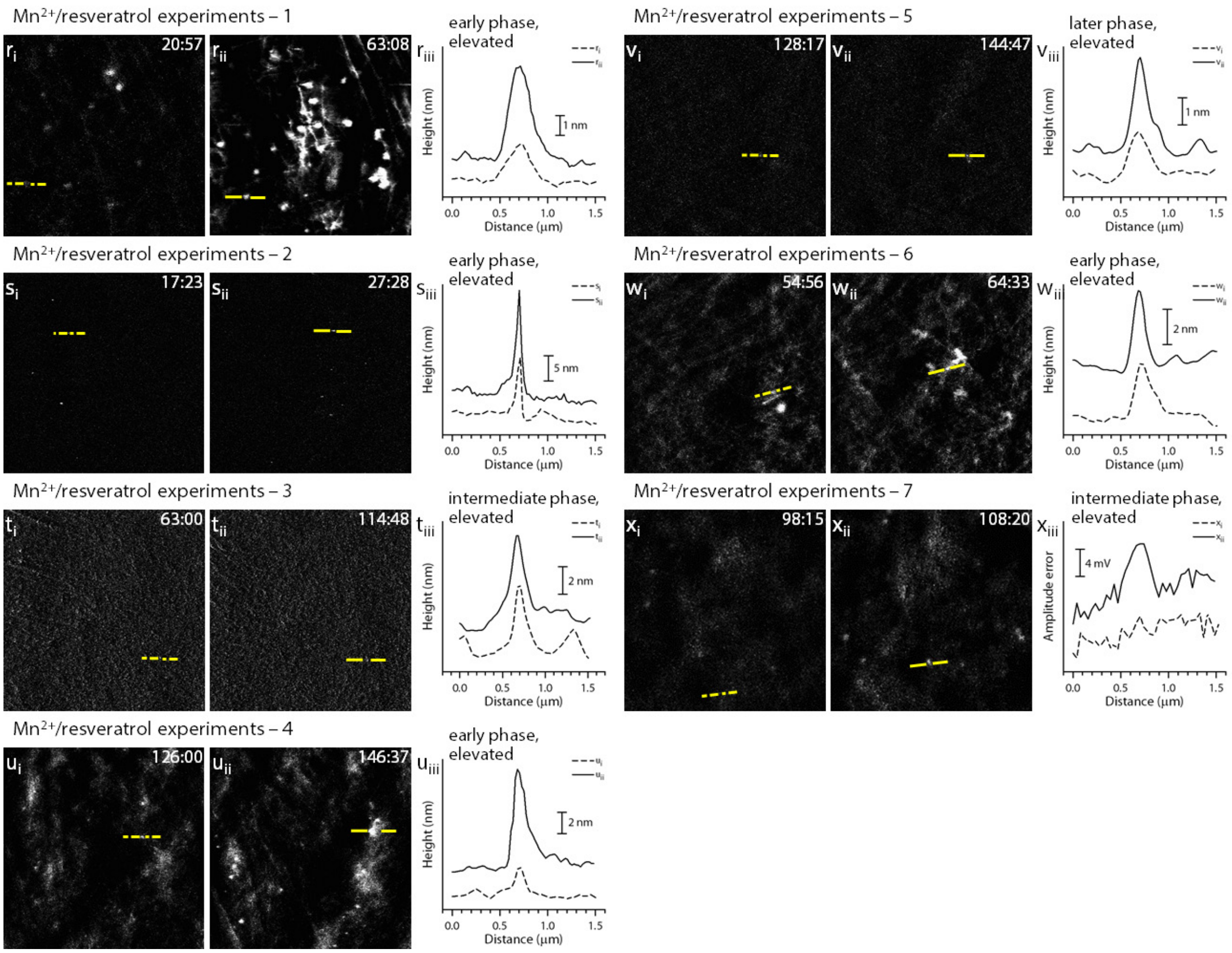

Fig. S12. Representative AFM images for resveratrol/Mn^{2+}-activated MCF-7 cells. Within the 10-μm imaging zones on each cell, features consistent with the findings presented in the main text were observed in 8 out of 10 independent experiments. Seven of these instances are displayed herein, while the remaining case is detailed in the main text. The protrusions exhibited an increase in height rather than in diameter. These images were obtained by Tapping Mode and processed by Hadamard product. Image size: 10 μm × 10 μm.

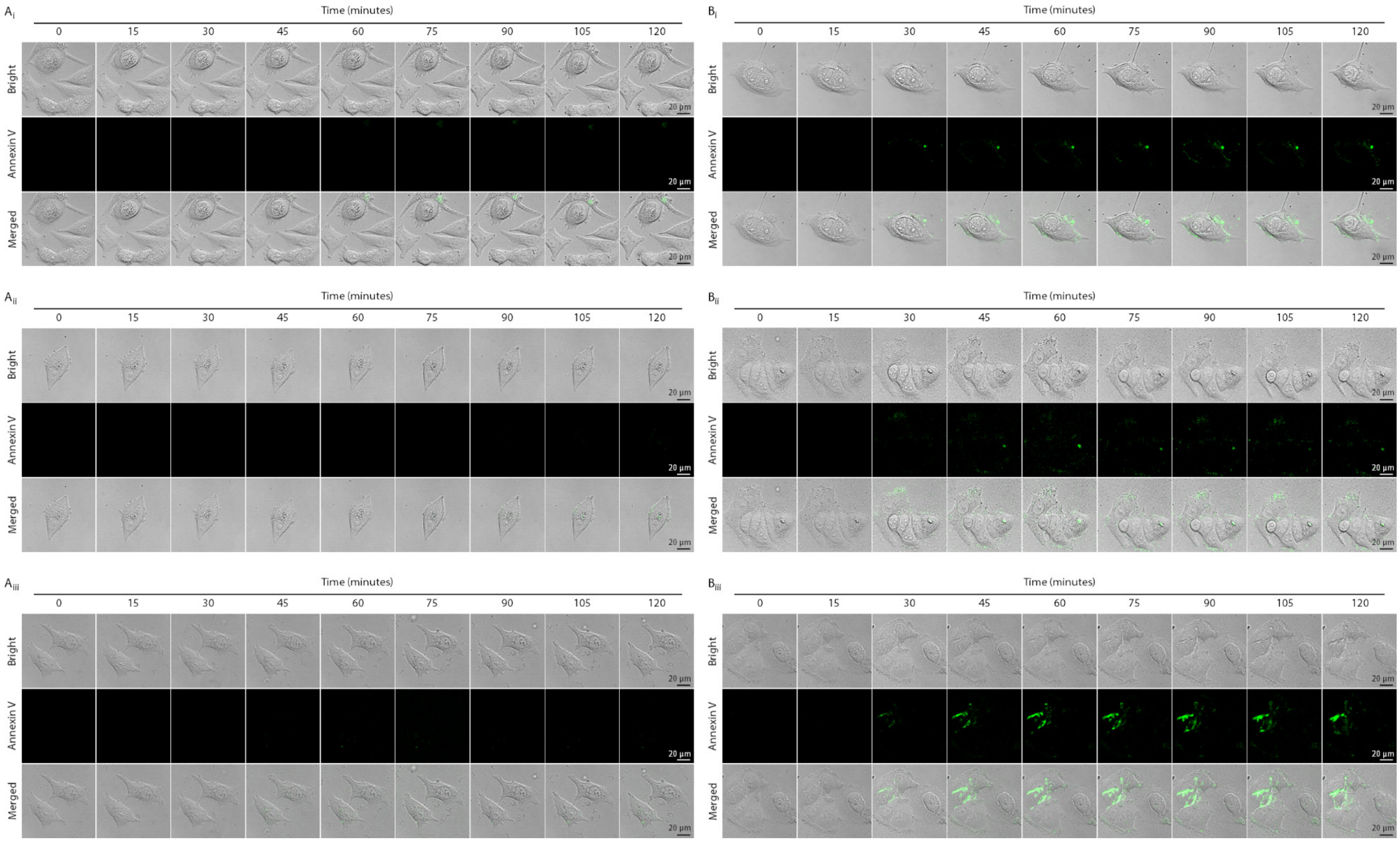

Ai
Time (minutes)
0
15
30
45
60
75
90
105
120
Bright
Annexin V
Merged
20 µm
Bi
Aii
Bii
Aiii
Biii

Fig. S13. Confocal microscopic images of resveratrol/Mn^{2+}-treated MCF-7. MCF-7 cells were treated with (**A**) 10 μM resveratrol alone or (**B**) 50 μM Mn^{2+} and 10 μM resveratrol in PBS supplemented with Alexa Fluor® 488-conjugated Annexin V (green). Experimental conditions were identical to those in fig. S8. Each condition, with and without Mn^{2+}, were examined in three independent experiments, yielding consistent and reproducible results that further support the findings of fig. S8.

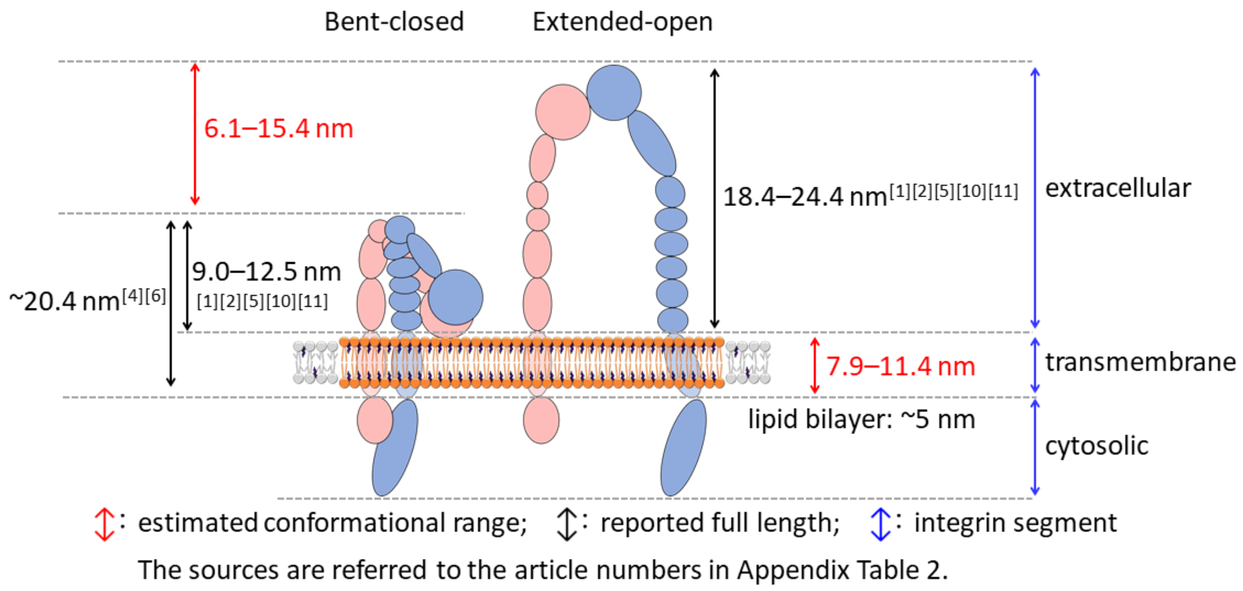

Fig. S14. Estimated heights of integrin αvβ3 in bent-closed and extended-open states based on X-ray structures summarized in table S2. Live-cell AFM measurements (3.7–25.4 nm; Supporting Information, fig. S7) align with ectodomain lengths from crystallography (9.0–24.4 nm). Ceramide-enriched domains protrude only ~1 nm above the bilayer (*71*, *72*). As tapping-mode AFM detects only extracellular features, these height changes likely reflect integrin ectodomain conformational dynamics rather than ceramide clustering or intracellular structures. The superscripted citation numbers correspond to the numbered literature articles in table S2.

Data S1. Entries for the 90 trackable nanodomains. This file documents the entries of protrusions in Fig. 4.

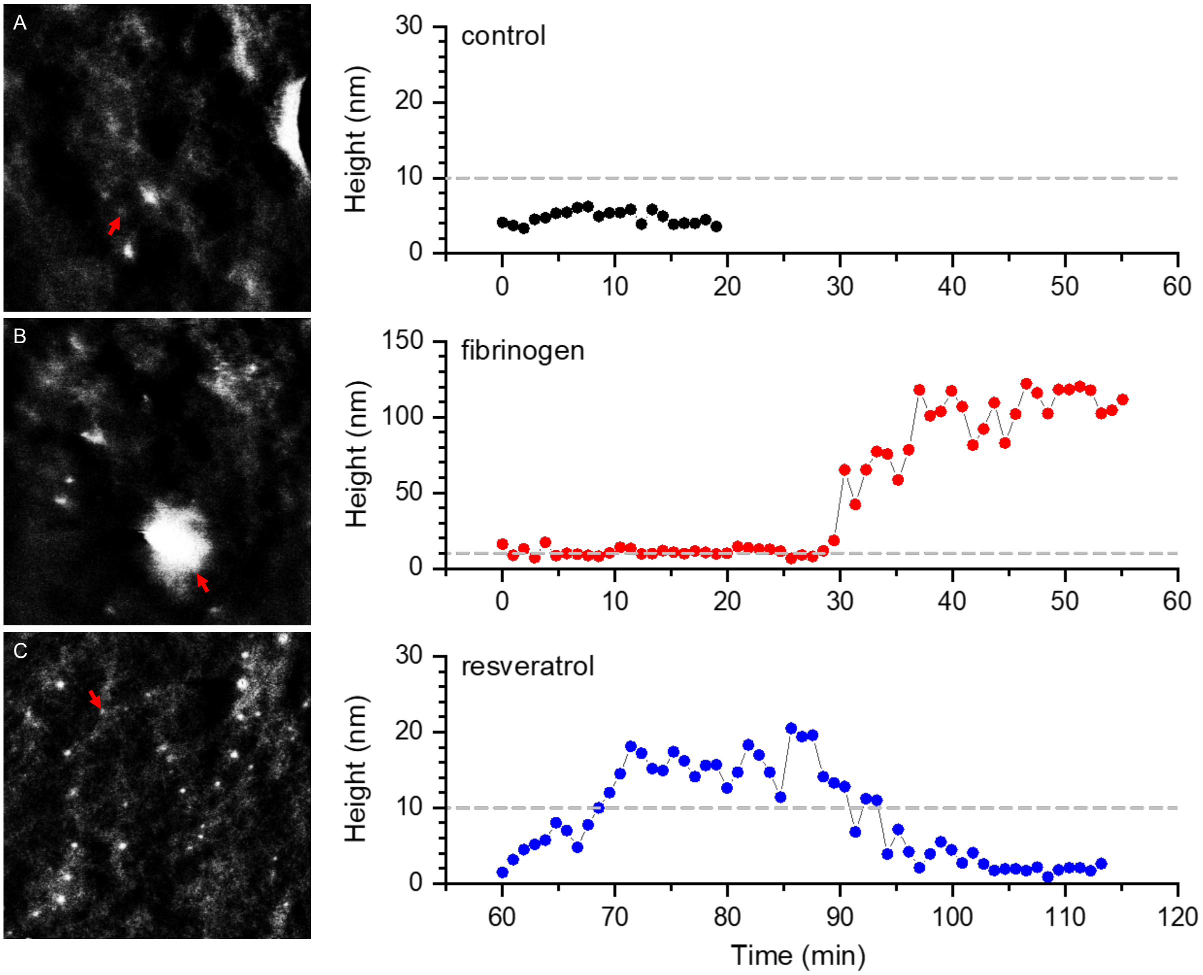

Movie S1. Height variation of membrane raft on live MCF-7 cell. Movie is presented in trajectories and height variation in (**A**) PBS, (**B**) PBS with 70 µM fibrinogen and (C) PBS with 50 µM Mn^{2+} and 10 µM resveratrol. The height of colored dots in the right plots are measured from the assigned arrows in the corresponding left images. The substantial variations of approximately 100 nm and 20 nm after administering of fibrinogen and resveratrol contrast with the control, where the variations are below 10 nm. The gray dash lines are reference height at 10 nm. scan rate: 28 sec/frame; image size: (A,B) 5 µm × 5 µm, (C) 10 µm × 10 µm. Other imaging conditions were the same as those of Fig. 2.

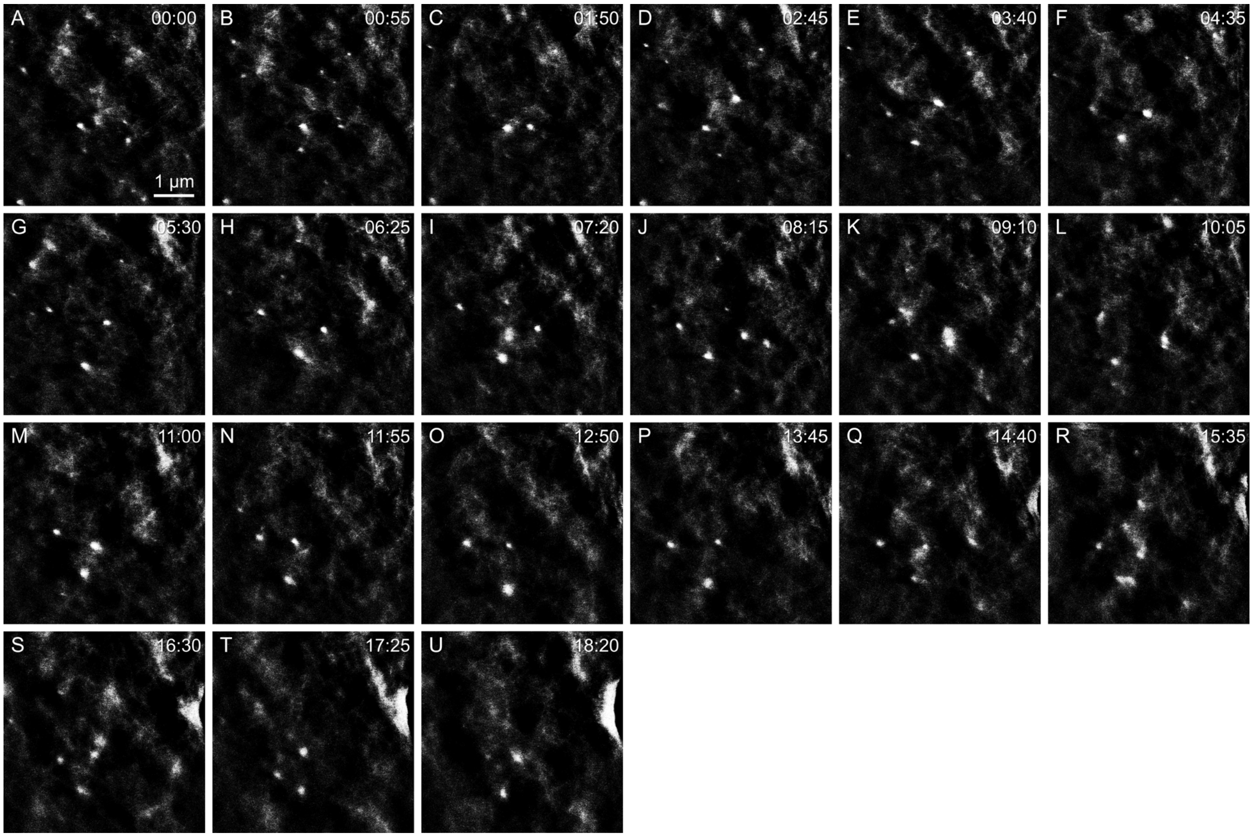

Movie S2. Dynamics of membrane rafts on a live MCF-7 cell in plain phosphate buffer. Images are subject to the operation of Hadamard product as described in fig. S2. Images A–E are presented as panels A_i–A_v of Fig. 3 in the main text. Supplementary Movie S2 is prepared from this image set. Distinct from those in figs. S1 and S2 are that images of figs. S3–S5 and Supplementary Movies S1–S4 were intentionally performed on areas without major cellular skeletons in the image area. Conditions: solution, blank PBS buffer; scan rate: 28 sec/frame; image size: 5 μm × 5 μm. Other conditions were the same as fig. S2.

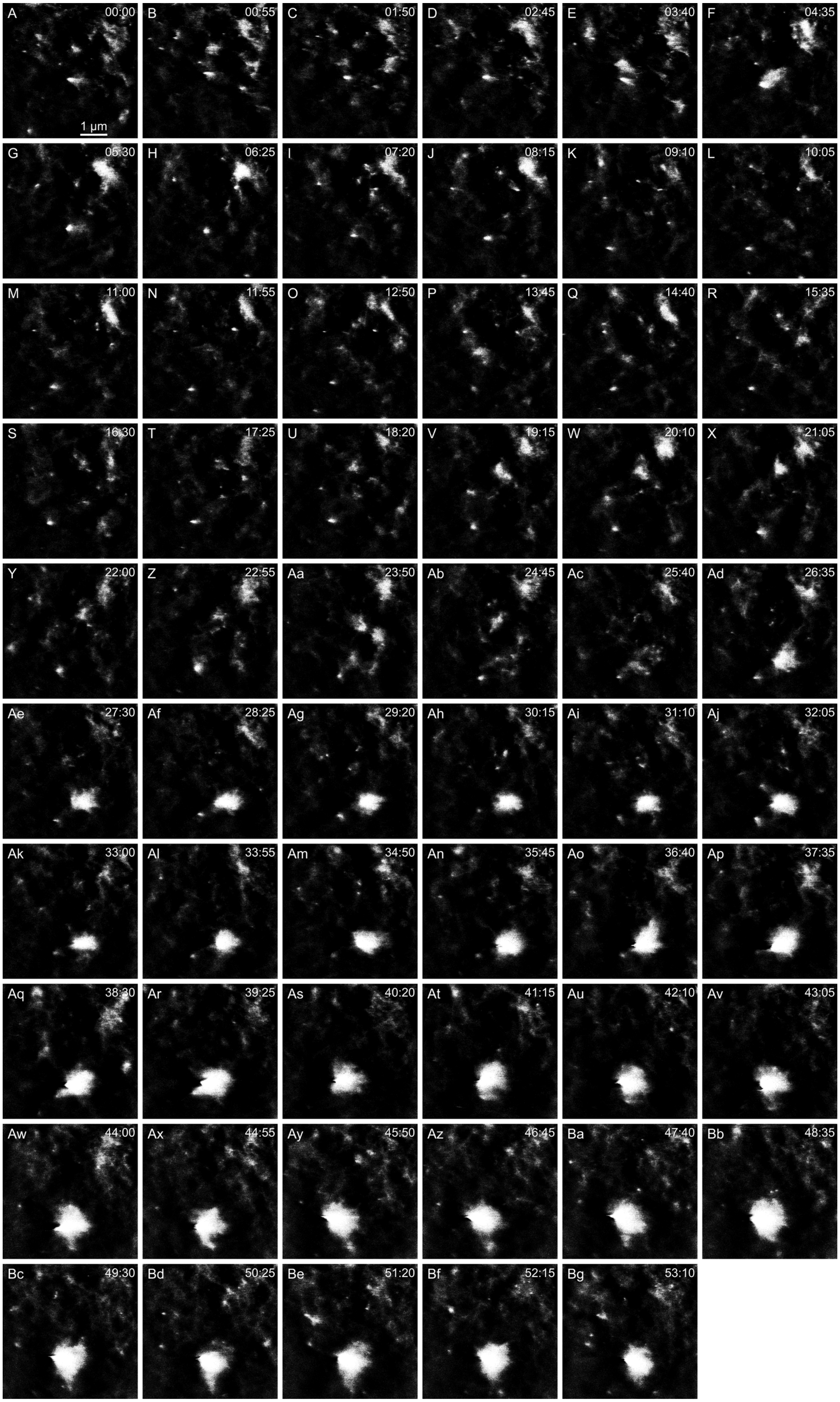
A 00:00
1 µm
B 00:55
C 01:50
D 02:45
E 03:40
F 04:35
G 05:30
H 06:25
I 07:20
J 08:15
K 09:10
L 10:05
M 11:00
N 11:55
O 12:50
P 13:45
Q 14:40
R 15:35
S 16:30
T 17:25
U 18:20
V 19:15
W 20:10
X 21:05
Y 22:00
Z 22:55
Aa 23:50
Ab 24:45
Ac 25:40
Ad 26:35
Ae 27:30
Af 28:25
Ag 29:20
Ah 30:15
Ai 31:10
Aj 32:05
Ak 33:00
Al 33:55
Am 34:50
An 35:45
Ao 36:40
Ap 37:35
Aq 38:30
Ar 39:25
As 40:20
At 41:15
Au 42:10
Av 43:05
Aw 44:00
Ax 44:55
Ay 45:50
Az 46:45
Ba 47:40
Bb 48:35
Bc 49:30
Bd 50:25
Be 51:20
Bf 52:15
Bg 53:10

Movie S3. Dynamic response of membrane rafts to fibrinogen on a live MCF-7 cell. Images are subject to the operation of Hadamard product as described in fig. S1. There are coagulated entities with ~1-μm in size after administering of fibrinogen for ~5 min (in panel G) and ~26 min (in panel Ad). Images Z, Ab, Ac, Ad, and Af are presented, respectively, as panels B_i–B_v of Fig. 3 in the main text. Supplementary Movie S3 is prepared from this image set. Conditions: solution, PBS with 70 μM fibrinogen; scan rate: 28 sec/frame; image size: 5 μm × 5 μm. Other conditions were the same as fig. S2.

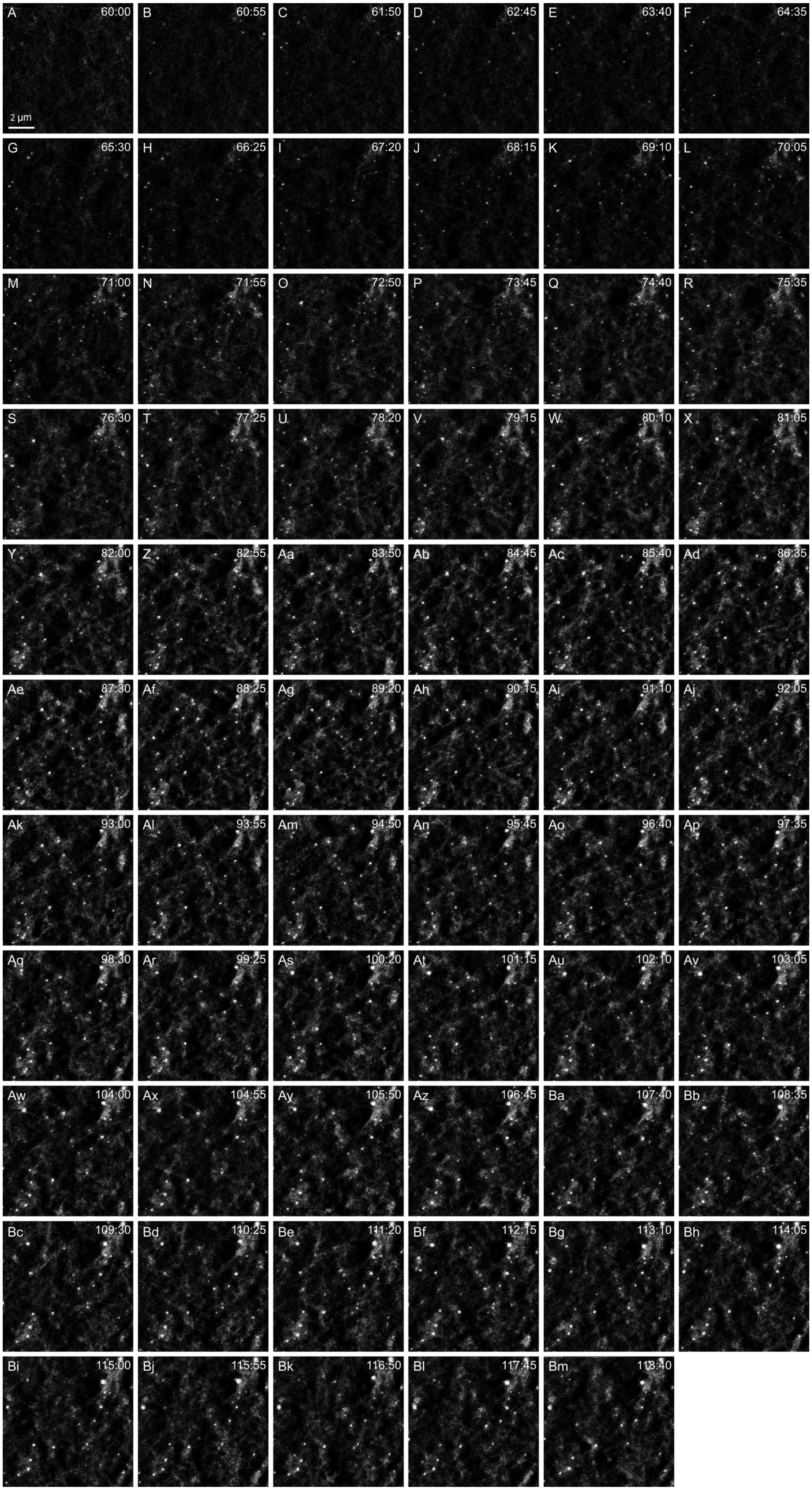
A 60:00
B 60:55
C 61:50
D 62:45
E 63:40
F 64:35
2 µm
G 65:30
H 66:25
I 67:20
J 68:15
K 69:10
L 70:05
M 71:00
N 71:55
O 72:50
P 73:45
Q 74:40
R 75:35
S 76:30
T 77:25
U 78:20
V 79:15
W 80:10
X 81:05
Y 82:00
Z 82:55
Aa 83:50
Ab 84:45
Ac 85:40
Ad 86:35
Ae 87:30
Af 88:25
Ag 89:20
Ah 90:15
Ai 91:10
Aj 92:05
Ak 93:00
Al 93:55
Am 94:50
An 95:45
Ao 96:40
Ap 97:35
Aq 98:30
Ar 99:25
As 100:20
At 101:15
Au 102:10
Av 103:05
Aw 104:00
Ax 104:55
Ay 105:50
Az 106:45
Ba 107:40
Bb 108:35
Bc 109:30
Bd 110:25
Be 111:20
Bf 112:15
Bg 113:10
Bh 114:05
Bi 115:00
Bj 115:55
Bk 116:50
Bl 117:45
Bm 118:40

Movie S4. Dynamic response of membrane rafts to resveratrol/Mn^{2+} on a live MCF-7 cell. Images are subject to the operation of Hadamard product as described in fig. S1. Presented images were acquired 60 min after introducing Mn^{2+}/resveratrol because it took ~1 hr for Mn^{2+} to activate integrin $\alpha_V\beta_3$ toward resveratrol (please see fig. S8). The density and heights (~15 nm) of the white protrusions are larger than those with plain PBS buffer (figs. S2 and S3). The white features clustered and formed plateaus in the upper right and lower left, yet individual protrusions did not fuse as those ~1-μm ones in the case of fibrinogen (fig. S4). Images K, Q, W, An, and As are presented, respectively, as panels C_i–C_v of Fig. 3 in the main text. Supplementary Movie S4 is prepared from this image set. Conditions: solution, PBS with 50 μM Mn^{2+} and 10 μM resveratrol; scan rate: 28 sec/frame; image size: 10 μm × 10 μm. Other conditions are the same as fig. S2.

Movie S5. Time-resolved sectional profiles of protrusions on the live cell membrane treated with resveratrol/Mn^{2+}. This movie is prepared from images for Fig. 4 in the main text. Conditions: solution, PBS with 50 μM Mn^{2+} and 10 μM resveratrol; scan rate: 28 sec/frame; image size: 10 μm × 10 μm. Conditions are the same as Fig. 4.

Table S1. Sizes of protruded features before and after introducing fibrinogen and Mn^{2+}/resveratrol.

	introduced stimulant[a]	isolated features[b]		features[c] after treatment[d]	
		size range	avg	size range	avg
before treatment	---	50−250 nm	150 nm	---	---
after treatment	fibrinogen	70−550 nm	238 nm	0.8−2.0 μm	1.3 μm
	Mn^{2+}/resveratrol	40−820 nm	250 nm	0.7−3.3 μm[e]	1.8 μm

[a] The experimental conditions are the same as Fig. 3 in which MCF-7 cells under AFM observation were in PBS buffers with stimulants of (1) 70 μM fibrinogen or (2) 50 μM Mn^{2+} and 10 μM resveratrol.

[b] An *isolated feature* is referred to a single protruded nanodomain that is identifiable, as pointed by pink arrows in the images displayed below.

[c] The appearance of the newly formed features is shown below in fig. S9B (red) and S9C (yellow) after treated with fibrinogen and Mn^{2+}/resveratrol, respectively. For the feature (the red circle) in fig. S9B, isolated protruded nanodomains were coagulated. Individual protruded nanodomains are indistinguishable and unassignable. For the newly formed features (yellow traces) in fig. S9C, several protruded nanodomains converged and developed terraces in which there were taller and stiffer protrusions.

[d] The features were observed starting from *ca.* 30 min and 60 min after introducing fibrinogen and resveratrol to MCF-7, respectively. Throughout the course of AFM observation, isolated protruded nanodomains were always present.

[e] To report the size for irregularly shaped features, employed herein is Feret diameter with the spacing between the two parallel tangential lines restricting the feature perpendicular to that direction. (https://en.wikipedia.org/wiki/Feret_diameter)

Table S2. Summary of estimated heights of integrin $\alpha_V\beta_3$ in different conformational states from x-ray crystallographic data

	literature*	Segment	Methods	Results (estimated)[#]		Δh (nm)
				Bent-closed	Extended-open	
1	Crystal structure of the extracellular segment of integrin $\alpha_V\beta_3$. *Science* **294**, 339–345 (2001).	extracellular	X-ray	~12.3 nm	~20.5 nm	~8.2
2	Novel pure $\alpha_V\beta_3$ integrin antagonists that do not induce receptor extension, prime the receptor, or enhance angiogenesis at low concentrations. *ACS Pharmacol. Transl. Sci.* **2**, 387–401 (2019).	extracellular/ extracellular + ligand	X-ray (including ligand)	~20.4 nm	~30.6 nm	~10.2
			EM (without ligand)	~12.5 nm	~20.9nm	~8.4
3	Crystal structure of the extracellular segment of integrin $\alpha_V\beta_3$ in complex with an Arg–Gly–Asp Ligand. *Science* **296**, 151–155 (2002).	extracellular + RGD	X-ray (including RGD)	~15.0 nm	~20.5 nm	~5.5
4	Crystal structure of the complete integrin $\alpha_V\beta_3$ ectodomain plus an α/β transmembrane fragment. *J. Cell Biol.* **186**, 589–600 (2009).	extracellular + transmembrane (TM)	X-ray	NA	~20.4 nm	NA
5	The therapeutic antibody LM609 selectively inhibits ligand binding to human $\alpha_V\beta_3$ integrin via steric hindrance. *J. Biol. Chem.* **292**, 1732–1739.e5 (2017)	extracellular	X-ray	~12.4 nm	~18.4nm	~6.0
			Neg-stain EM	~12.0 nm	~20.0 nm	~8.0
6	$\alpha_V\beta_3$ Integrin crystal structures and their functional implications. *Biochemistry* **51**, 8814–8828 (2012).	extracellular + transmembrane (TM)	X-ray	~20.4 nm	NA	NA
7	Structural basis of the differential binding of engineered knottins to integrins $\alpha_V\beta_3$ and $\alpha_5\beta_1$. *Structure* **27**, 1443–1451.e6 (2019).	extracellular + Knottin	X-ray (including ligand)	17.0 ~18.0 nm	~20.4 nm	2.4 ~3.4
8	Atomic basis for the species-specific inhibition of α_V integrins by monoclonal antibody 17E6 is revealed by the crystal structure of $\alpha_V\beta_3$-17E6 Fab complex. *J. Biol. Chem.* **289**, 13801–13809 2014).	extracellular + 17E6 Fab	X-ray (including ligand)	~13.4 nm	~26.7nm	~13.3
			EM	~14.0 nm	NA	NA
9	Structural basis for pure antagonism of integrin $\alpha_V\beta_3$ by a high-affinity form of fibronectin. *Nat. Struct. Mol Biol.* **21**, 383–388 (2014).	extracellular + fibronectin	X-ray (including ligand)	NA	~20.4nm	NA
10	Three-dimensional EM structure of the ectodomain of integrin $\alpha_V\beta_3$ in a complex with fibronectin. *J. Cell Biol.* **168**, 1109–1118 (2005)	extracellular + fibronectin	Neg-stain EM	~9 nm	~20 nm	~11
11	Molecular dynamics simulations of forced unbending of integrin $\alpha_V\beta_3$. *PLoS Comput. Biol.* **7**, e1001086 (2011).	extracellular	Steered MD simulation	~11.4 nm	~24.4 nm	~13.0

*The integrins were (articles 1-10) experimentally activated by Mn^{2+} and (article 11) by Ca^{2+} and Mg^{2+} for the simulation one.
[#]The values were estimated based on the reported crystallographic parameters.

REFERENCES AND NOTES

1. B. Diaz-Rohrer, K. R. Levental, I. Levental, Rafting through traffic: Membrane domains in cellular logistics. *Biochim. Biophys. Acta Biomembr.* **1838**, 3003–3013 (2014).

2. B. B. Diaz-Rohrer, K. R. Levental, K. Simons, I. Levental, Membrane raft association is a determinant of plasma membrane localization. *Proc. Natl. Acad. Sci. U.S.A.* **111**, 8500–8505 (2014).

3. K. Simons, D. Toomre, Lipid rafts and signal transduction. *Nat. Rev. Mol. Cell Biol.* **1**, 31–39 (2000).

4. A. Kusumi, T. K. Fujiwara, N. Morone, K. J. Yoshida, R. Chadda, M. Xie, R. S. Kasai, K. G. N. Suzuki, Membrane mechanisms for signal transduction: The coupling of the meso-scale raft domains to membrane-skeleton-induced compartments and dynamic protein complexes. *Semin. Cell Dev. Biol.* **23**, 126–144 (2012).

5. E. Sezgin, I. Levental, S. Mayor, C. Eggeling, The mystery of membrane organization: Composition, regulation and roles of lipid rafts. *Nat. Rev. Mol. Cell Biol.* **18**, 361–374 (2017).

6. K. Simons, E. Ikonen, Functional rafts in cell membranes. *Nature* **387**, 569–572 (1997).

7. S. L. Veatch, N. Rogers, A. Decker, S. A. Shelby, The plasma membrane as an adaptable fluid mosaic. *Biochim. Biophys. Acta Biomembr.* **1865**, 184114 (2023).

8. D. Lingwood, K. Simons, Lipid rafts as a membrane-organizing principle. *Science* **327**, 46–50 (2010).

9. T. Harayama, H. Riezman, Understanding the diversity of membrane lipid composition. *Nat. Rev. Mol. Cell Biol.* **19**, 281–296 (2018).

10. M. Kinoshita, N. Matsumori, Inimitable impacts of ceramides on lipid rafts formed in artificial and natural cell membranes. *Membranes* **12**, 727 (2022).

11. L. C. Silva, R. F. M. de Almeida, B. M. Castro, A. Fedorov, M. Prieto, Ceramide-domain formation and collapse in lipid rafts: Membrane reorganization by an apoptotic lipid. *Biophys. J.* **92**, 502–516 (2007).

12. L. J. Pike, Rafts defined: A report on the Keystone symposium on lipid rafts and cell function. *J. Lipid Res.* **47**, 1597–1598 (2006).

13. H. Heerklotz, Triton promotes domain formation in lipid raft mixtures. *Biophys. J.* **83**, 2693–2701 (2002).

14. M. Robinson, C. T. Filice, D. M. McRae, Z. Leonenko, Atomic force microscopy and other scanning probe microscopy methods to study nanoscale domains in model lipid membranes. *Adv. Phys. X* **8**, 2197623 (2023).

15. I. Levental, K. R. Levental, F. A. Heberle, Lipid rafts: Controversies resolved, mysteries remain. *Trends Cell Biol.* **30**, 341–353 (2020).

16. K. G. N. Suzuki, A. Kusumi, Refinement of singer-nicolson fluid-mosaic model by microscopy imaging: Lipid rafts and actin-induced membrane compartmentalization. *Biochim. Biophys. Acta Biomembr.* **1865**, 184093 (2023).

17. M. Lorizate, O. Terrones, J. A. Nieto-Garai, I. Rojo-Bartolomé, D. Ciceri, O. Morana, J. Olazar-Intxausti, A. Arboleya, A. Martin, M. Szynkiewicz, M. Calleja-Felipe, J. Bernardino de la Serna, F. X. Contreras, Super-resolution microscopy using a bioorthogonal-based cholesterol probe provides unprecedented capabilities for imaging nanoscale lipid heterogeneity in living cells. *Small Methods* **5**, e2100430 (2021).

18. H. Kemmoku, K. Takahashi, K. Mukai, T. Mori, K. M. Hirosawa, F. Kiku, Y. Uchida, Y. Kuchitsu, Y. Nishioka, M. Sawa, T. Kishimoto, K. Tanaka, Y. Yokota, H. Arai, K. G. N. Suzuki, T. Taguchi, Single-molecule localization microscopy reveals STING clustering at the trans-Golgi network through palmitoylation-dependent accumulation of cholesterol. *Nat. Commun.* **15**, 220 (2024).

19. M. Maekawa, G. D. Fairn, Complementary probes reveal that phosphatidylserine is required for the proper transbilayer distribution of cholesterol. *J. Cell Sci.* **128**, 1422–1433 (2015).

20. J. D. Nickels, S. Chatterjee, C. B. Stanley, S. Qian, X. L. Cheng, D. A. A. Myles, R. F. Standaert, J. G. Elkins, J. Katsaras, The in vivo structure of biological membranes and evidence for lipid domains. *PLoS Biol.* **15**, e2002214 (2017).

21. G. L. Nicolson, G. Ferreira de Mattos, The fluid–mosaic model of cell membranes: A brief introduction, historical features, some general principles, and its adaptation to current information. *Biochim. Biophys. Acta Biomembr.* **1865**, 184135 (2023).

22. A. J. Borst, Z. M. James, W. N. Zagotta, M. Ginsberg, F. A. Rey, F. DiMaio, M. Backovic, D. Veesler, The therapeutic antibody LM609 selectively inhibits ligand binding to human $\alpha_V\beta_3$ integrin via steric hindrance. *Structure* **25**, 1732–1739.e5 (2017).

23. J. Li, Y. Fukase, Y. Shang, W. Zou, J. M. Muñoz-Félix, L. Buitrago, J. van Agthoven, Y. Zhang, R. Hara, Y. Tanaka, R. Okamoto, T. Yasui, T. Nakahata, T. Imaeda, K. Aso, Y. Zhou, C. Locuson, D. Nesic, M. Duggan, J. Takagi, R. D. Vaughan, T. Walz, K. Hodivala-Dilke, S. L. Teitelbaum, M. A. Arnaout, M. Filizola, M. A. Foley, B. S. Coller, Novel pure $\alpha_V\beta_3$ integrin antagonists that do not induce receptor extension, prime the receptor, or enhance angiogenesis at low concentrations. *ACS Pharmacol. Transl.* **2**, 387–401 (2019).

24. C. Eggeling, A. Honigmann, Closing the gap: The approach of optical and computational microscopy to uncover biomembrane organization. *Biochim. Biophys. Acta Biomembr.* **1858**, 2558–2568 (2016).

25. A. L. Duncan, W. Pezeshkian, Mesoscale simulations: An indispensable approach to understand biomembranes. *Biophys. J.* **122**, 1883–1889 (2023).

26. J. Fan, M. Sammalkorpi, M. Haataja, Lipid microdomains: Structural correlations, fluctuations, and formation mechanisms. *Phys. Rev. Lett.* **104**, 118101 (2010).

27. J. Fan, M. Sammalkorpi, M. Haataja, Influence of nonequilibrium lipid transport, membrane compartmentalization, and membrane proteins on the lateral organization of the plasma membrane. *Phys. Rev. E* **81**, 011908 (2010).

28. J. Fan, M. Sammalkorpi, M. Haataja, Formation and regulation of lipid microdomains in cell membranes: Theory, modeling, and speculation. *FEBS Lett.* **584**, 1678–1684 (2010).

29. M. Krieg, G. Fläschner, D. Alsteens, B. M. Gaub, W. H. Roos, G. J. L. Wuite, H. E. Gaub, C. Gerber, Y. F. Dufrêne, D. J. Müller, Atomic force microscopy-based mechanobiology. *Nat. Rev. Phys.* **1**, 41–57 (2019).

30. M. Li, N. Xi, L. Liu, Hierarchical micro-/nanotopography for tuning structures and mechanics of cells probed by atomic force microscopy. *IEEE Trans. Nanobioscience* **20**, 543–553 (2021).

31. L. Zhang, L. Zhao, P.-K. Ouyang, P. Chen, Insight into the role of cholesterol in modulation of morphology and mechanical properties of CHO-K1 cells: An in situ AFM study. *Front. Chem. Sci. Eng.* **13**, 98–107 (2019).

32. M. Shibata, H. Watanabe, T. Uchihashi, T. Ando, R. Yasuda, High-speed atomic force microscopy imaging of live mammalian cells. *Biophys. Physicobiol.* **14**, 127–135 (2017).

33. Y. Shan, H. Wang, The structure and function of cell membranes examined by atomic force microscopy and single-molecule force spectroscopy. *Chem. Soc. Rev.* **44**, 3617–3638 (2015).

34. J. Iturri, A. Weber, A. Moreno-Cencerrado, M. dM Vivanco, R. Benítez, S. Leporatti, J. L. Toca-Herrera, Resveratrol-induced temporal variation in the mechanical properties of MCF-7 breast cancer cells investigated by atomic force microscopy. *Int. J. Mol. Sci.* **20**, 3275 (2019).

35. X. Wu, X. Yu, C. Chen, C. Chen, Y. Wang, D. Su, L. Zhu, Fibrinogen and tumors. *Front. Oncologia* **14**, 1393599 (2024).

36. H.-Y. Lin, L. Lansing, J.-M. Merillon, F. B. Davis, H.-Y. Tang, A. Shih, X. Vitrac, S. Krisa, T. Keating, H. J. Cao, J. Bergh, S. Quackenbush, P. J. Davis, Integrin $\alpha_V\beta_3$ contains a receptor site for resveratrol. *FASEB J.* **20**, 1742–1744 (2006).

37. Y. Ho, Z. Li, Y. J. Shih, Y.-R. Chen, K. Wang, J. Whang-Peng, H.-Y. Lin, P. J. Davis, Integrin $\alpha_V\beta_3$ in the mediating effects of dihydrotestosterone and resveratrol on breast cancer cell proliferation. *Int. J. Mol. Sci.* **21**, 2906 (2020).

38. X. Pang, X. He, Z. Qiu, H. Zhang, R. Xie, Z. Liu, Y. Gu, N. Zhao, Q. Xiang, Y. Cui, Targeting integrin pathways: Mechanisms and advances in therapy. *Signal. Transduct. Target. Ther.* **8**, 1 (2023).

39. T. M. Odrljin, C. G. Haidaris, N. B. Lerner, P. J. Simpson-Haidaris, Integrin $\alpha_V\beta_3$-mediated endocytosis of immobilized fibrinogen by A549 lung alveolar epithelial cells. *Am. J. Respir. Cell Mol. Biol.* **24**, 12–21 (2001).

40. V. Reyhani, P. Seddigh, B. Guss, R. Gustafsson, L. Rask, K. Rubin, Fibrin binds to collagen and provides a bridge for $\alpha_V\beta_3$ integrin-dependent contraction of collagen gels. *Biochem. J.* **462**, 113–123 (2014).

41. D. P. Ly, K. M. Zazzali, S. A. Corbett, De novo expression of the integrin $\alpha_5\beta_1$ regulates αvβ3-mediated adhesion and migration on fibrinogen. *J. Biol. Chem.* **278**, 21878–21885 (2003).

42. M. M. Pesho, K. Bledzka, L. Michalec, C. S. Cierniewski, E. F. Plow, The specificity and function of the metal-binding sites in the integrin β_3 A-domain. *J. Biol. Chem.* **281**, 23034–23041 (2006).

43. P. Zhang, T. Ozdemir, C.-Y. Chung, G. P. Robertson, C. Dong, Sequential binding of $\alpha_V\beta_3$ and ICAM-1 determines fibrin-mediated melanoma capture and stable adhesion to CD11b/CD18 on neutrophils. *J. Immunol.* **186**, 242–254 (2011).

44. M. Rolli, E. Fransvea, J. Pilch, A. Saven, B. Felding-Habermann, Activated integrin $\alpha_V\beta_3$ cooperates with metalloproteinase MMP-9 in regulating migration of metastatic breast cancer cells. *Proc. Natl. Acad. Sci. U.S.A.* **100**, 9482–9487 (2003).

45. P. J. Davis, S. A. Mousa, V. Cody, H.-Y. Tang, H.-Y. Lin, Small molecule hormone or hormone-like ligands of integrin $\alpha_V\beta_3$: Implications for cancer cell behavior. *Horm. Cancer* **4**, 335–342 (2013).

46. H. J. Garrigues, L. K. DeMaster, Y. E. Rubinchikova, T. M. Rose, KSHV attachment and entry are dependent on $\alpha_V\beta_3$ integrin localized to specific cell surface microdomains and do not correlate with the presence of heparan sulfate. *Virology.* **464-465**, 118–133 (2014).

47. T. Gianni, V. Gatta, G. Campadelli-Fiume, $\alpha_V\beta_3$-Integrin routes herpes simplex virus to an entry pathway dependent on cholesterol-rich lipid rafts and dynamin2. *Proc. Natl. Acad. Sci. U.S.A.* **107**, 22260–22265 (2010).

48. S. Chakraborty, M. ValiyaVeettil, S. Sadagopan, N. Paudel, B. Chandran, c-Cbl-mediated selective virus-receptor translocations into lipid rafts regulate productive Kaposi's sarcoma-associated herpesvirus infection in endothelial cells. *J. Virol.* **85**, 12410–12430 (2011).

49. F. Orsini, A. Cremona, P. Arosio, P. A. Corsetto, G. Montorfano, A. Lascialfari, A. M. Rizzo, Atomic force microscopy imaging of lipid rafts of human breast cancer cells. *Biochim. Biophys. Acta Biomembr.* **1818**, 2943–2949 (2012).

50. J. A. Peruzzi, T. F. Gunnels, H. I. Edelstein, P. Lu, D. Baker, J. N. Leonard, N. P. Kamat, Enhancing extracellular vesicle cargo loading and functional delivery by engineering protein-lipid interactions. *Nat. Commun.* **15**, 5618 (2024).

51. B. Kollmitzer, P. Heftberger, R. Podgornik, J. F. Nagle, G. Pabst, Bending rigidities and interdomain forces in membranes with coexisting lipid domains. *Biophys. J.* **108**, 2833–2842 (2015).

52. Y. Tian, Y. Wu, L. Liu, L. He, J. Gao, L. Zhou, F. Yu, S. Yu, H. Wang, The structural characteristics of mononuclear-macrophage membrane observed by atomic force microscopy. *J. Struct. Biol.* **206**, 314–321 (2019).

53. C. Bernard, A. R. Carotenuto, N. M. Pugno, M. Fraldi, L. Deseri, Modelling lipid rafts formation through chemo-mechanical interplay triggered by receptor–ligand binding. *Biomech. Model. Mechanobiol.* **23**, 485–505 (2024).

54. W. Chen, J. Lou, J. Hsin, K. Schulten, S. C. Harvey, C. Zhu, Molecular dynamics simulations of forced unbending of integrin $\alpha_V\beta_3$. *PLoS Comput. Biol.* **7**, e1001086 (2011).

55. H. K. Gaikwad, S. V. Jaswandkar, K. S. Katti, A. Haage, D. R. Katti, Molecular basis of conformational changes and mechanics of integrins. *Philos. Trans. R. Soc. A* **381**, 20220243 (2023).

56. Y. Chen, H. Lee, H. Tong, M. Schwartz, C. Zhu, Force regulated conformational change of integrin $\alpha_V\beta_3$. *Matrix Biol.* **60-61**, 70–85 (2017).

57. F. Lu, L. Zhu, T. Bromberger, J. Yang, Q. Yang, J. Liu, E. F. Plow, M. Moser, J. Qin, Mechanism of integrin activation by talin and its cooperation with kindlin. *Nat. Commun.* **13**, 2362 (2022).

58. J. Li, M. H. Jo, J. Yan, T. Hall, J. Lee, U. López-Sánchez, S. Yan, T. Ha, T. A. Springer, Ligand binding initiates single-molecule integrin conformational activation. *Cell* **187**, 2990–3005.e17 (2024).

59. O. Schussler, J. C. Chachques, M. Alifano, Y. Lecarpentier, Key roles of RGD-recognizing integrins during cardiac development, on cardiac cells, and after myocardial infarction. *J. Cardiovasc. Transl. Res.* **15**, 179–203 (2022).

60. C. Gest, U. Joimel, L. Huang, L.-L. Pritchard, A. Petit, C. Dulong, C. Buquet, C.-Q. Hu, P. Mirshahi, M. Laurent, F. Fauvel-Lafève, L. Cazin, J.-P. Vannier, H. Lu, J. Soria, H. Li, R. Varin, C. Soria, Rac3 induces a molecular pathway triggering breast cancer cell aggressiveness: Differences in MDA-MB-231 and MCF-7 breast cancer cell lines. *BMC Cancer* **13**, 63 (2013).

61. T. K. Harden, S. N. Hicks, J. Sondek, Phospholipase C isozymes as effectors of Ras superfamily GTPases. *J. Lipid Res.* **50**, S243–S248 (2009).

62. J. Xu, X. Huang, Lipid metabolism at membrane contacts: Dynamics and functions beyond lipid homeostasis. *Front. Cell Dev. Biol.* **8**, 615856 (2020).

63. S. L. Veatch, P. Cicuta, P. Sengupta, A. Honerkamp-Smith, D. Holowka, B. Baird, Critical fluctuations in plasma membrane vesicles. *ACS Chem. Biol.* **3**, 287–293 (2008).

64. J. Gómez, F. Sagués, R. Reigada, Nonequilibrium patterns in phase-separating ternary membranes. *Phys. Rev. E* **80**, 011920 (2009).

65. J. Chen, Q. Zou, Large-range high-speed dynamic-mode atomic force microscope imaging: Adaptive tapping towards minimal force. *Nanotechnology* **34**, 455503 (2023).

66. J. M. Green, A. Zheleznyak, J. Chung, F. P. Lindberg, M. Sarfati, W. A. Frazier, E. J. Brown, Role of cholesterol in formation and function of a signaling complex involving $\alpha_V\beta_3$, integrin-associated protein (CD47), and heterotrimeric G proteins. *J. Cell Biol.* **146**, 673–682 (1999).

67. A. Cormier, M. G. Campbell, S. Ito, S. Wu, J. Lou, J. Marks, J. L. Baron, S. L. Nishimura, Y. Cheng, Cryo-EM structure of the $\alpha_V\beta_8$ integrin reveals a mechanism for stabilizing integrin extension. *Nat. Struct. Mol. Biol.* **25**, 698–704 (2018).

68. X.-P. Xu, E. Kim, M. Swift, J. W. Smith, N. Volkmann, D. Hanein, Three-dimensional structures of full-length, membrane-embedded human $\alpha_{IIb}\beta_3$ integrin complexes. *Biophys. J.* **110**, 798–809 (2016).

69. R. I. Litvinov, M. Mravic, H. Zhu, J. W. Weisel, W. F. DeGrado, J. S. Bennett, Unique transmembrane domain interactions differentially modulate integrin $\alpha_V\beta_3$ and $\alpha_{IIb}\beta_3$ function. *Proc. Natl. Acad. Sci. U.S.A.* **116**, 12295–12300 (2019).

70. Y. Chen, Z. Li, F. Kong, L. A. Ju, C. Zhu, Force-regulated spontaneous conformational changes of integrins $\alpha_5\beta_1$ and $\alpha_V\beta_3$. *ACS Nano* **18**, 299–313 (2024).

71. I. Johnston, L. J. Johnston, Ceramide promotes restructuring of model raft membranes. *Langmuir* **22**, 11284–11289 (2006).

72. S. Chiantia, N. Kahya, J. Ries, P. Schwille, Effects of ceramide on liquid-ordered domains investigated by simultaneous AFM and FCS. *Biophys. J.* **90**, 4500–4508 (2006).
